# Profiling of gallbladder carcinoma reveals distinct miRNA profiles and activation of STAT1 by the tumor suppressive miRNA-145-5p

**DOI:** 10.1038/s41598-019-40857-3

**Published:** 2019-03-18

**Authors:** Benjamin Goeppert, Felicia Truckenmueller, Alessandro Ori, Valerie Fritz, Thomas Albrecht, Angelika Fraas, Dominique Scherer, Rosa González Silos, Carsten Sticht, Norbert Gretz, Arianeb Mehrabi, Melanie Bewerunge-Hudler, Stefan Pusch, Justo Lorenzo Bermejo, Peter Dietrich, Peter Schirmacher, Marcus Renner, Stephanie Roessler

**Affiliations:** 1 Institute of Pathology, University Hospital Heidelberg, Heidelberg, Germany; 2 Leibniz Institute on Aging - Fritz Lipmann Institute (FLI), Jena, Germany; 3 Institute of Biochemistry, Emil-Fischer Zentrum, Friedrich-Alexander-University Erlangen-Nürnberg, Erlangen, Germany; 4 Institute of Medical Biometry and Informatics, University Hospital Heidelberg, Heidelberg, Germany; 5 Center of Medical Research, University Hospital Mannheim, Mannheim, Germany; 6 Department of General Visceral and Transplantation Surgery, University Hospital Heidelberg, Heidelberg, Germany; 7 Genomics and Proteomics Core Facility, German Cancer Research Center (DKFZ), Heidelberg, Germany; 8 Heidelberg University Hospital, Institute of Pathology, Department of Neuropathology, Heidelberg, Germany and Clinical Cooperation Unit Neuropathology, German Cancer Research Center, Heidelberg, Germany; 9 Department of Medicine, Friedrich-Alexander-University Erlangen-Nürnberg, Erlangen, Germany

## Abstract

Gallbladder carcinoma (GBC) is a biliary tract cancer with few treatment options and poor prognosis. Radical surgery is the only potentially curative treatment option but most patients diagnosed with GBC are unresectable. Thus, there is a great need for the development of new treatment options including targeted therapy. Here, we aimed at identifying deregulated miRNAs and affected pathways involved in GBC development and progression. We performed global miRNA profiling of 40 GBC and 8 normal gallbladder tissues and identified large differences with 30% of miRNAs being differentially expressed (false discovery rate: FDR < 0.001). We found 24 miRNAs to be differentially regulated in GBC with poor outcome (p < 0.05) of which miR-145-5p was the most downregulated miRNA. Overexpression of miR-145-5p significantly reduced cell proliferation and colony formation. Gene expression analysis of cells expressing miR-145-5p mimics revealed activation of the Signal transducer and activator of transcription 1 (STAT1) signaling pathway which is mainly tumor suppressive. Furthermore, the activation of STAT1 by miR-145-5p was specifically observed in gallbladder carcinoma and cholangiocarcinoma but not in hepatocellular carcinoma cells. The Protein Tyrosine Phosphatase Receptor Type F (PTPRF) is downregulated upon miR-145 expression and may be involved in STAT1 regulation. In addition, we found that the STAT1-regulated protein IRF7 is downregulated in GBC compared to normal gallbladder tissue and low IRF7 expression is associated with significantly lower overall survival of GBC patients. Thus, this study identified GBC patient subgroups and provides new mechanistic insights in the tumor suppressive function of miR-145-5p leading to activation of STAT1 signaling.

## Introduction

Gallbladder carcinoma (GBC) is an understudied disease that together with intra- (iCCA) and extrahepatic cholangiocarcinoma (eCCA) constitutes the group of biliary tract cancers (BTC). GBC is a rare disease with an incidence of less than 2/100,000 worldwide but the incidence varies strongly between geographical areas^1^. In Northern Europe, incidence rates of less than 1/100,000 have been reported, whereas, among Native American and South American women the incidence is as high as 23/100,000^2,3^. Due to its late and non-specific symptoms, GBC is mostly diagnosed at advanced stages with therefore limited treatment options and poor prognosis. Overall survival of early GBC (stage I) is close to 90%, however, the majority of GBCs are diagnosed at advanced stages (stage III or IV) with 5-year survival rates <10%^4^. Radical surgery is the only potentially curative treatment option and the combination therapy of Cisplatin and Gemcitabine seems to be currently the most effective chemotherapeutic agent^5,6^. However, chemotherapeutic approaches extent the overall survival rate by a few months only and to date, there is no targeted treatment option established.

The main risk factors for GBC include cholelithiasis (presence of gallstones), chronic inflammation of the gallbladder wall, female gender, age, ethnicity, genetic predisposition, diabetes and obesity^7^. GBC mostly evolves in a background of symptomatic gallstones and chronic inflammation of the gallbladder. However, only 1–3% of patients with gallstones develop GBC and on the other hand 10–15% of patients with GBC show no evidence of gallstones^8^. Thus, additional specific genetic or epigenetic factors are required for gallbladder carcinogenesis. In contrary to more common human tumors, there is relatively limited information about the molecular changes involved in the development of GBC. In GBC identified mutations and genomic alterations involve the tumor suppressor genes TP53, ARID1A and SMAD4 as well as the oncogenes ERBB2/HER2, CDKN2A/B, KRAS, PIK3CA and EGFR^9–12^. These genomic alterations open a window for targeted treatment opportunities, however, only a subset of patients is affected by these mutations. Therefore, it is important to study epigenetic alterations and functional pathways involved in GBC development and progression.

MicroRNAs (miRNAs) are small non-coding RNAs that can bind to the 3′ untranslated regions (UTRs) of target mRNAs thereby either preventing translation or leading to the degradation of target mRNAs^13^. Deregulation of miRNAs has been shown to support a wide range of diseases including cancer^14–16^. Recently, miR-1, miR-145-5p, miR-20a and miR-155 have been identified to be involved in GBC development and progression^17–19^. Chang *et al*. performed an *in vitro* screen of metastatic miRNAs and assayed the top 10 miRNA candidates in a cohort of 67 Chinese patients^17^. Due to the known function in other inflammation-associated carcinomas, Kono *et al*. analyzed miR-155 in 26 Japanese GBC patients and Letelier *et al*. performed miRNA microarray analysis in 8 Chilean GBC tissues^18,19^. However, none of these studies analyzed the global effect of miRNA perturbation on downstream targets in a large Western European cohort and due to the higher prevalence; most studies have been performed in cohorts of South American or Asian ethnicity which exhibit distinct clinicopathological and molecular features^20,21^.

In the present study, we recruited a large German cohort and identified multiple miRNAs which are associated with GBC patient outcome. Thus, GBC patient subgroups with distinct miRNA expression patterns exist. We showed that miR-145-5p expression significantly reduced cell proliferation and colony formation. In contrary, ectopic expression of miR-575 increased cell proliferation rate and colony formation capacity. By whole genome mRNA expression profiling of cell lines expressing miR-145-5p, we found the STAT1 signaling pathway to be the most prominently altered pathway. Activation of STAT1 by miR-145-5p was only induced in biliary tract cell lines but not in hepatocellular carcinoma cell lines. In addition, miR-145-5p expression led to inhibition of the phosphatase PTPRF in biliary tract but not hepatocellular carcinoma cell lines. Thus, loss of miR-145-5p expression in GBC patients may lead to decreased STAT1 signaling.

## Results

### MiRNAs associated with patient outcome in GBC

**Figure 1:**
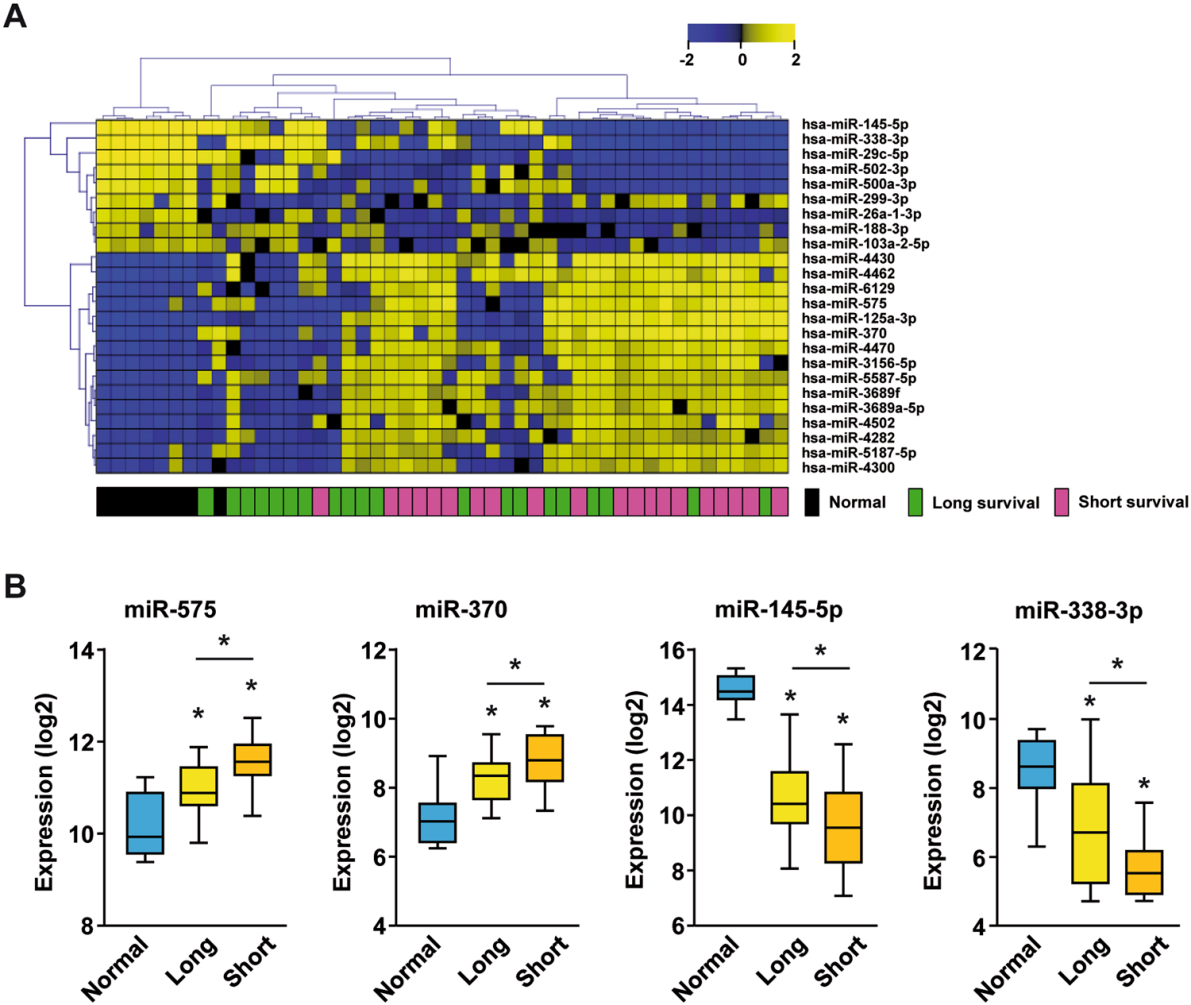
MiRNA profiling of normal gallbladder and GBC tissues. (**A**) Hierarchical clustering of 40 GBC and 8 non-neoplastic gallbladder tissues using Euclidean distance of 24 survival-associated miRNAs. **(B)** Relative Expression (log2) of miR-575, miR-370, miR-145-5p and miR-338-3p in non-neoplastic gallbladder (N = 8), GBC patients with long survival (>17.2 months; N = 20) or short survival (<17.2 months; N = 20). * indicates p < 0.05.

We performed miRNA profiling of 40 gallbladder tumors tissues and 8 non-neoplastic gallbladder tissues (normal tissue). The clinicopathological data of these 40 GBC patients are summarized in Table 1. This GBC cohort included mainly advanced disease patients with 90% UICC III or UICC IV and accordingly overall poor outcome with 5-year survival of 25.3% (Fig. S1). Median GBC patient survival was 17.2 months and this cutoff was chosen to separate patients into two survival groups. Importantly, comparison of the short and long survival groups revealed that none of the clinical parameters, including UICC staging, perineural, lymphatic invasion, vascular invasion, and pre-existing hepatobiliary tract disease, were associated with patient outcome (Table 1). Thus, patient survival of this relatively homogenous GBC cohort is not significantly associated with any of these clinicopathological parameters. Global miRNA profiling of the 40 GBC and 8 non-neoplastic gallbladder tissues revealed clear differences with 608 out of 2006 miRNAs being differentially expressed (FDR < 0.001). Thus, 30% of all miRNAs tested were deregulated in tumor tissues compared to normal gallbladder. To select key survival-associated miRNAs, we restricted our analysis to miRNAs whose expression was significantly differentially expressed between non-neoplastic gallbladder and GBC samples (FDR < 0.001) with significant differences between long and short survivors (median survival cut-off; p < 0.05). This revealed 24 miRNAs to be differentially expressed between survival groups with 8 miRNAs being down- and 16 miRNAs being upregulated in GBC, respectively (Table S1). Supervised hierarchical clustering of these 24 miRNAs showed that normal gallbladder tissues clustered together and GBC cases with poor outcome tended to cluster separately from long survival cases, suggesting two molecularly distinct GBC patient subgroups (Fig. 1A). The two most downregulated miRNAs, miR-145-5p and miR-338-3p and the top upregulated miRNAs, miR-575 and miR-370, showed gradual expression changes between the normal gallbladder tissues, the GBC long survival group and the short survival group (Fig. 1B). Thus, we identified 24 miRNAs that were altered during GBC tumor development and were differentially expressed in patients with poor outcome revealing distinct GBC patient subgroups.

**Table 1:** Patient characteristics.

GBC patients		All cases	Short survival	Long survival	p-value
		**40**	**20**	**20**	
Age	median (years)	70.1	70.6	67.8	0.386^a^
Sex	female	27	12	15	0.500^b^
	male	13	8	5	
UICC stage	UICC I	1	0	1	0.825^b^
	UICC II	2	1	1	
	UICC III	19	11	8	
	UICC IV	8	5	3	
	UICC NA	10	3	7	
Primary tumour	pT1	3	1	2	0.688^b^
	pT2	17	7	10	
	pT3	15	9	6	
	pT4	5	3	2	
Lymph nodes	pN0	9	2	7	0.184^b^
	pN1	12	7	5	
	pNx	19	11	8	
Metastasis	M0	34	16	18	0.661^b^
	M1	6	4	2	
Perineural invasion	Pn0	29	13	16	0.480^b^
	Pn1	11	7	4	
Lymphatic invasion	L0	24	10	14	0.333^b^
	L1	16	10	6	
Vascular invasion	V0	34	16	18	0.661^b^
	V1	6	4	2	
Resection	R0	15	6	9	0.227 ^b^
	R1	12	6	6	
	R2	6	5	1	
	Rx	7	3	4	
Pre-existing hepatobiliary tract disease	yes	20	13	7	0.113^b^
	no	20	7	13	

^a^T-test; ^b^Fisher’s exact test; missing data was omitted.

### MiR-145-5p acts tumor suppressive and miR-575 oncogenic in cell lines

**Figure 2:**
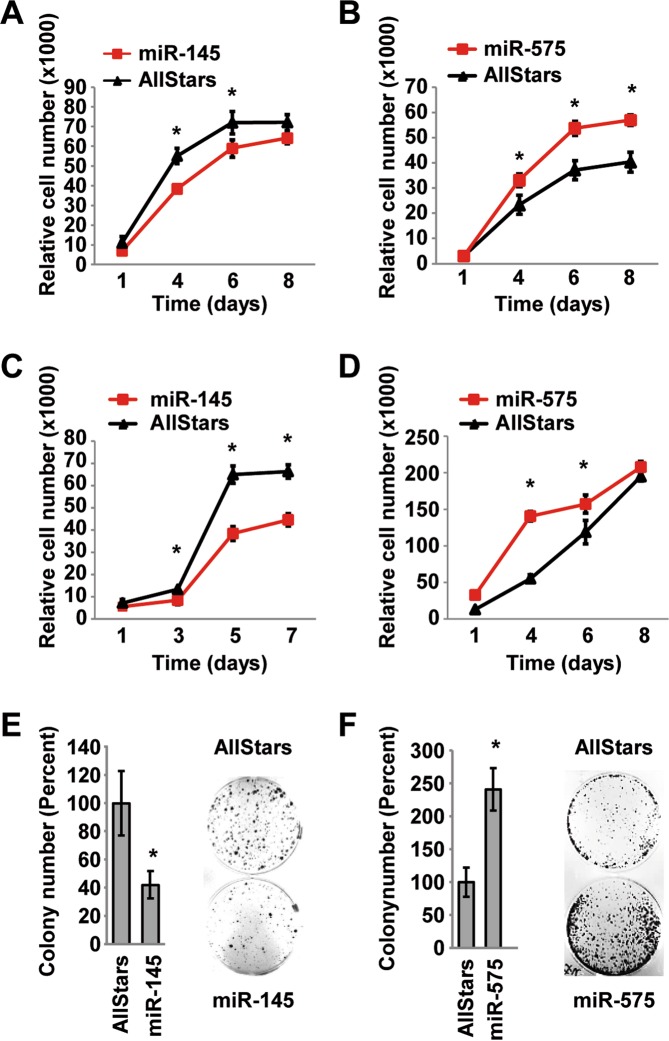
MiR-145-5p functions as a tumor suppressor and miR-575 as an oncogene in BTC cell lines. (**A**,**C)** miR-145-5p mimic overexpression in EGI-1 or TFK-1 cells leads to reduced cell viability, **(B**,**D)** whereas, miR-575 mimic overexpression in EGI-1 or TFK-1 leads to increased cell viability, respectively. **(E)** Colony formation capacity is reduced upon miR-145-5p expression and **(F)** increased upon miR-575 expression in TFK-1 cells. Representative results of three independent experiments are shown. * indicates p < 0.05.

Next, we validated the miRNA expression of the two top up- and downregulated miRNAs by qRT-PCR and functionally analyzed them (Fig. S2). We performed cell viability and colony formation assays of biliary tract cell lines overexpressing miR-145-5p, miR-338-3p, miR-575, miR-370 or AllStars control. We did not observe consistent effects of miR-338-3p or miR-370 expression on cell viability in EGI-1 and TFK-1, two different BTC cell lines (Fig. S3). However, transfection with miR-145-5p reduced, whereas, miR-575 increased cell viability of the EGI-1 cells (Fig. 2A,B). Similar results were obtained with the cell line TFK-1 (Fig. 2C,D). Colony formation assay of TFK-1 cells consistently showed that miR-145-5p reduced single cell growth (Fig. 2E), whereas, miR-575 increased colony formation (Fig. 2F). EGI-1 cells did not form cell colonies and therefore, no results were obtained. These results were in line with the patient expression profiles in which miR-145-5p was downregulated and miR-575 upregulated in GBC tumor tissue. In conclusion, miR-145-5p exhibited tumor suppressive and miR-575 oncogenic functions in cell culture experiments.

### MiR-145-5p expression leads to activation of STAT1 signaling

**Figure 3:**
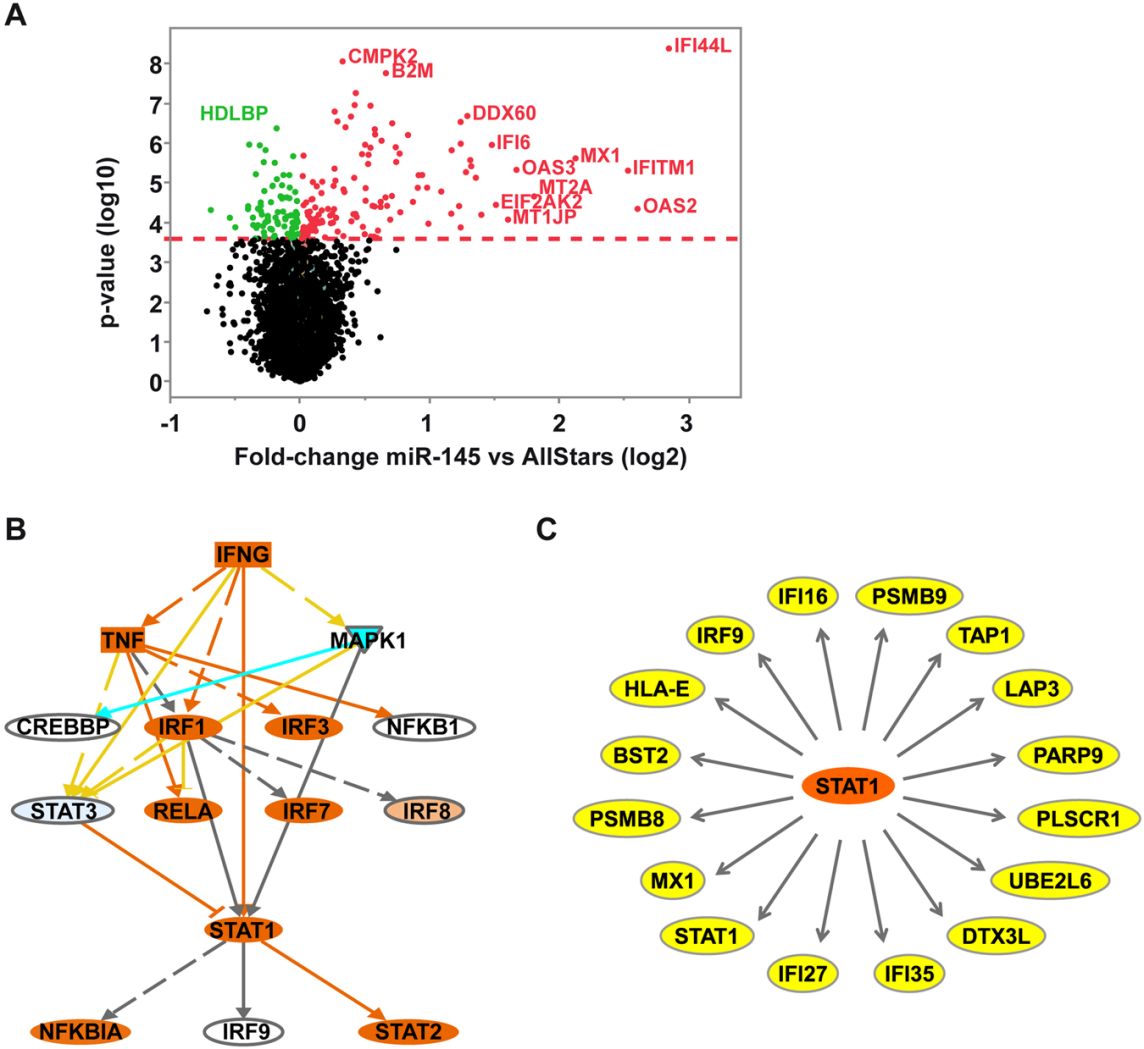
MiR-145-5p expression induces activation of the STAT1 pathway. (**A)** The volcano plot shows mRNA genes significantly deregulated in TFK-1 cells upon miR-145-5p expression. Green and red dots indicate significantly downregulated and upregulated genes, respectively. The red dashed line indicates a false positive rate of α = 0.05 with FDR correction. **(B)** Pathway analysis reveals upstream regulators of the gene profiles induced by miR-145-5p expression. **(C)** Sixteen (27.6%) of the 58 most upregulated genes after miR-145-5p expression are direct STAT1 target genes.

To identify target genes and pathways regulated by miR-145-5p, we transfected TFK-1 cells with AllStars control or miR-145-5p mimic and performed whole genome gene expression profiling. Volcano plot graphical view showed that most of the deregulated genes were upregulated (Fig. 3A). Class comparison identified 58 genes to be upregulated by miR-145-5p expression (adjusted p < 0.05; Table S2). In contrast, only two genes, CRIP1 and DANCR, were downregulated under the same cut-offs. Therefore, cut-offs were slightly relaxed, resulting in 31 downregulated genes (adjusted p < 0.1; Table S3). KEGG pathway analysis revealed that miR-145-5p expression induced multiple immune and inflammation related pathways in miR-145-5p expressing TFK-1 cells (Table S4). Furthermore, upstream Ingenuity Pathway Analysis suggested that the miR-145-5p gene signature was mainly regulated by immune modulatory transcriptions factors with STAT1 being at the centre of the network (Fig. 3B). Interestingly, among the 58 most upregulated genes 16 genes had been shown previously to be direct STAT1 targets (Table S2 and Fig. 3C)^22^. Next, we validated the observed induction of STAT1 mRNA expression and of STAT1 target genes by miR-145-5p using qRT-PCR in the biliary tract cell lines TFK-1 and EGI-1. Both cell lines, TFK-1 and EGI-1, similarly showed induction of STAT1 and its target genes MX1, IFI27 and PARP9 at mRNA levels (Fig. S4). In contrast, two hepatocellular carcinoma (HCC) cell lines, HuH1 and Hep3B, did not show a comparable induction of STAT1 mRNA levels or the three STAT1 target genes by miR-145-5p (Fig. S4). However, the inefficient STAT1 mRNA induction by miR-145-5p in the HCC cells was not caused by higher basal miR-145-5p levels as the expression was comparable to EGI-1 and TFK-1 cells (Fig. S5A). We also ruled out that this effect was the result of a lack of significant transfection efficiency: as shown by real time PCR, the transfection efficiency of the four cell lines was comparable (Fig. S5B).

**Figure 4:**
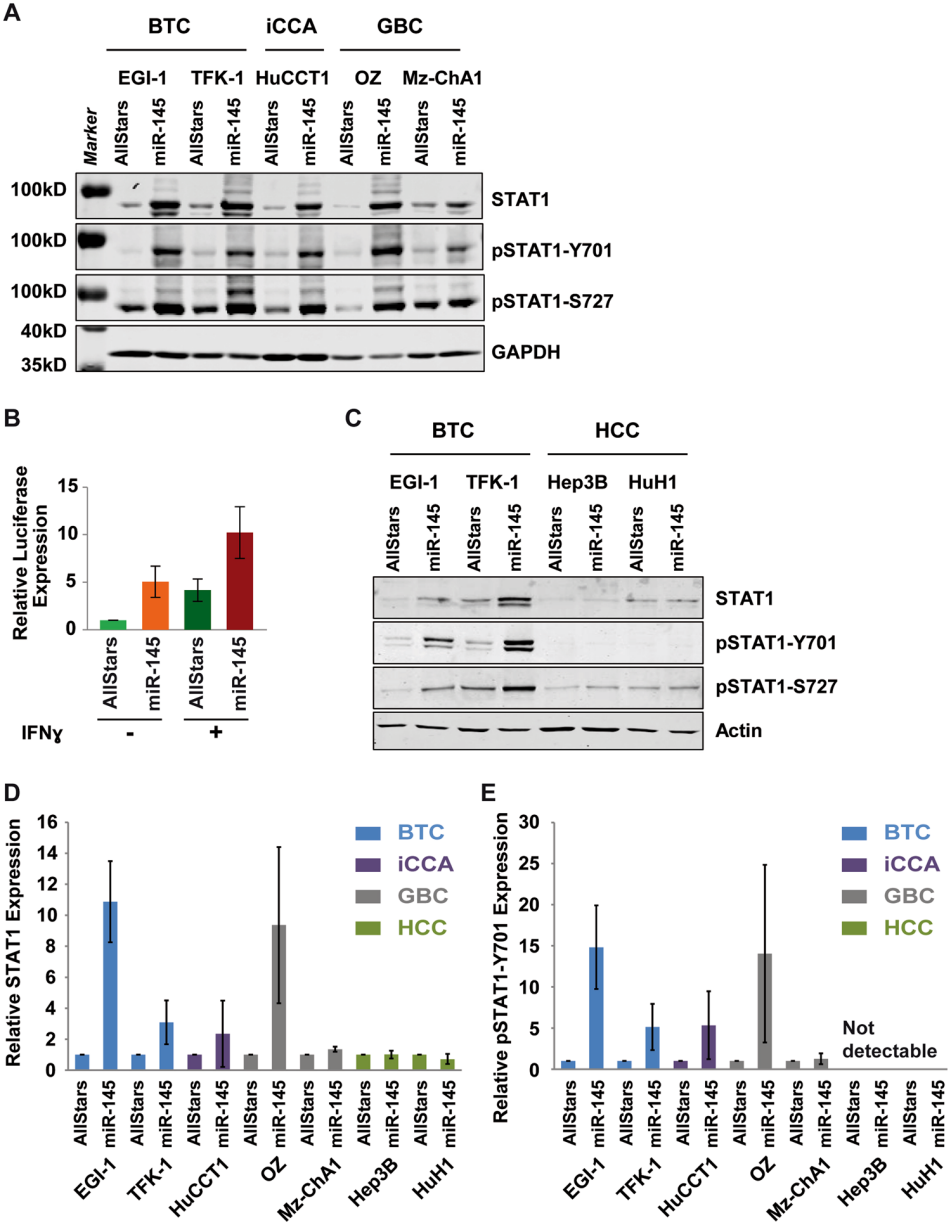
MiR-145-5p expression increases STAT1 phosphorylation and transcriptional activity. (**A**) Western blot analysis of total STAT1, STAT1 phosphorylated at tyrosine 701 (pSTAT1-Y701) or STAT1 phosphorylated at serine 727 (pSTAT1-S727) in BTC, iCCA and GBC cell lines transfected with control or miR-145-5p mimic, as indicated. GAPDH served as loading control. **(B)** Luciferase assay analysing STAT1 transcriptional activity using a GAS-luciferase reporter construct in TFK-1 cells expressing AllStars control or miR-145-5p. Prior harvesting cells were stimulated with or without 500 U/ml IFNγ for 8 h. Data represent mean ± SD of four independent biological experiments normalized to unstimulated AllStars control. **(C)** Western blot analysis of total STAT1, STAT1 phosphorylated at tyrosine 701 (pSTAT1-Y701) or STAT1 phosphorylated at serine 727 (pSTAT1-S727) in HCC cell lines transfected with control or miR-145-5p mimic, as indicated. Actin served as loading control. **(D**,**E)** Quantification of STAT1 and pSTAT1-Y701 expression by Western blot in BTC, iCCA, GBC and HCC cell lines, respectively. Data represent mean ± SD of three independent biological experiments normalized to AllStars control.

STAT1 signaling involves a positive feedback loop, whereby, STAT1 directly binds to its own promoter region^22,23^. To analyze whether increased STAT1 mRNA levels were caused by phosphorylation of STAT1 protein, we performed Western blot analysis. Expression of miR-145-5p in BTC, iCCA and GBC cell lines increased total STAT1 protein expression (Fig. 4A). Using antibodies specific to phosphorylation of Tyrosine 701 or of Serine 727 of STAT1 (pSTAT1-Y701 and pSTAT1-S727), we found that miR-145-5p did not only increase total STAT1 protein levels but also induced STAT1 protein phosphorylation. In addition, we tested if activation of STAT1 was a result of unspecific immunological activation by miRNAs. Therefore, we expressed miR-575 as an additional control. In contrast to miR-145-5p, miR-575 neither elevated total STAT1, phopho-STAT1-Y701 nor phospho-STAT1-S727 protein levels (Fig. S6A). Interestingly, the GBC cell line Mz-ChA1 exhibited no alteration of STAT1. Consistent with the human situation, the here used cell lines appear to be very heterogeneous. For example, a TP53 mutation was detected in Mz-ChA1 but not in OZ cells, suggesting different tumor driving events. Thus, miR-145-5p may induce STAT1 total protein levels and STAT1 phosphorylation in a cell line-dependent manner.

Next, we performed luciferase reporter assays to analyze STAT1 transcriptional activity. In the absence of IFNγ treatment miR-145-5p expression induced luciferase expression of a reporter construct containing 4x interferon-gamma activated sites (GAS elements) which are bound by STAT1 homo-dimers (Fig. 4B). IFNγ treatment induced GAS-luciferase expression and was further augmented upon miR-145-5p transfection in TFK-1 (Fig. 4B) and EGI-1 cell lines (Fig. S6B). However, STAT1 protein levels and also STAT1 phosphorylation at Y701 or S727 were unaltered in the HCC cell lines HuH1 and Hep3B (Fig. 4C). These results were quantified in three independent experiments (Fig. 4D,E). Thus, the activation of the STAT1 pathway by miR-145-5p appeared to be specific to biliary tract carcinoma cells.

### Expression of the two protein tyrosine phosphatases PTPRF and PTPRS is inhibited by miR-145-5p

To identify potential mediators of miR-145-5p induced activation of STAT1 signaling, we analyzed total and phosphorylated protein expression levels of JAK, TYK, PIAS and SOCS proteins and found that phospho-JAK2-Y107, phospho-TYK2, PIAS1, PIAS3, PIAS4, SOCS1 and SOCS3 were not altered upon miR-145-5p expression (Fig. S7). Phosphorylated JAK1 and PIAS2 could not be detected. Thus, it appeared that JAK, TYK, PIAS and SOCS proteins are not involved in miR-145-5p-mediated STAT1 activation. This suggests that miR-145-5p leads to a non-canonical STAT1 activation downstream of JAK, TYK, PIAS and SOCS proteins.

**Figure 5:**
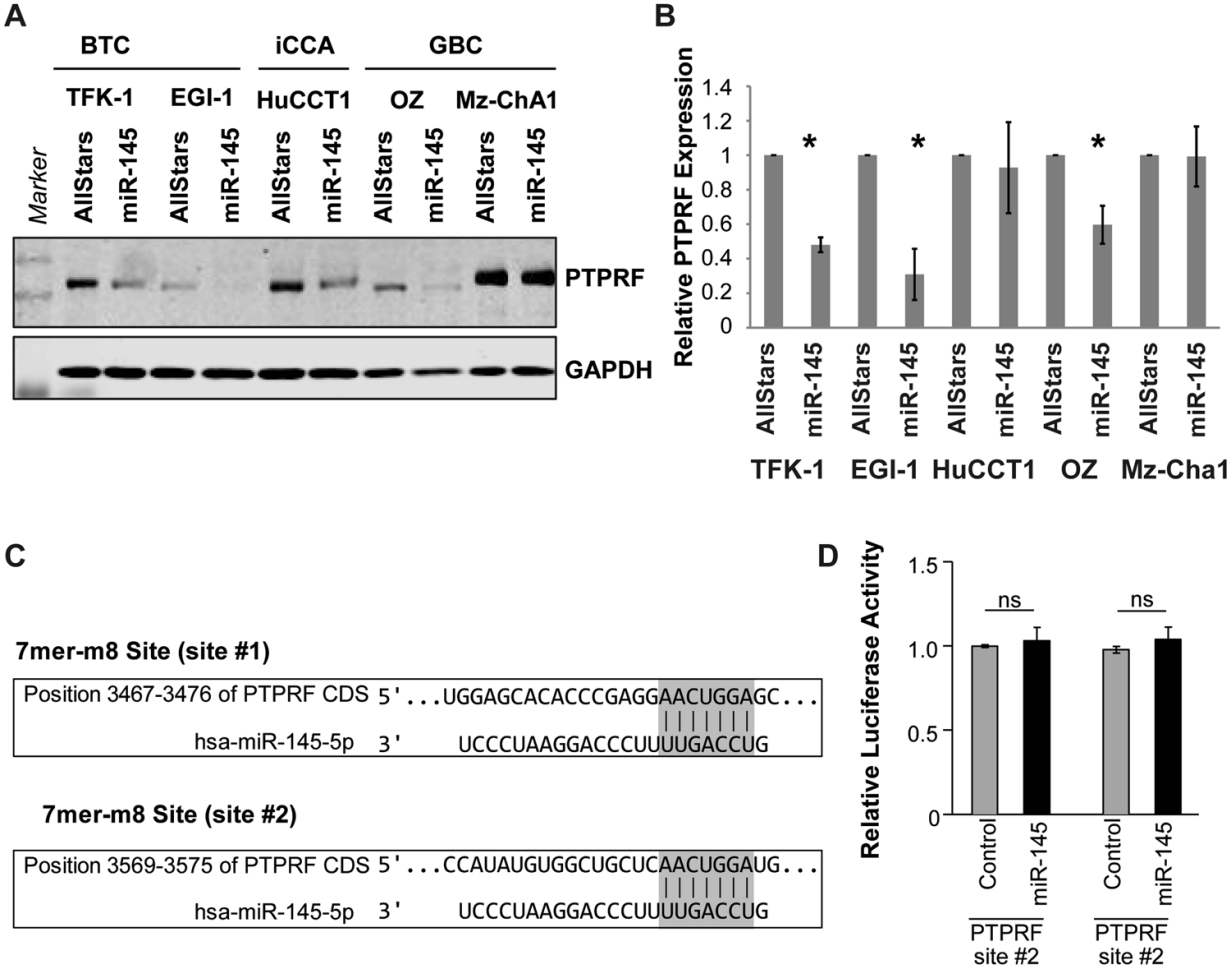
MiR-145-5p decreases PTPRF which leads to lower STAT1 phosphorylation through direct binding of STAT1. (**A)** Expression of PTPRF by Western blot upon Allstars or miR-145-5p expression in different cell lines. Shown is one representative experiment. **(B)** Quantification of PTPRF expression normalized by GAPDH expression relative to AllStars control of each cell line (N = 3). **(C)** Two non-canonical coding sequence (CDS) binding sites, site #1 and site #2, in the PTPRF mRNA for miR-145-5p were identified. **(D)** Relative luciferase activity of two PTPRF-CDS-binding-site-reporters was measured. Results for both potential non-canonical binding sites, site #1 and site #2, are depicted in AllStars (Control) and miR-145-5p-mimic (miR-145)-transfected cells. ns: non-significant.

Next, we analyzed genes downregulated upon miR-145-5p expression (Table S3). TargetScan (www.targetscan.org/) predicted CMTM4, GNAS and C12orf49 to be direct miR-145-5p targets (Table S3). Among the 10 most downregulated genes were the two Leukocyte Antigen Related (LAR) family protein tyrosine phosphatases PTPRF and PTPRS. Interestingly, it was shown previously that crosslinking of PTPRS on Gen2.2 cells delayed induction of both p-p38 and p-STAT1 and inhibited nuclear translocation of the STAT1-target IRF7^24,25^. We validated the downregulation of both genes at the mRNA level in TFK-1 cells (Fig. S8). PTPRS was not expressed in EGI-1 cells, whereas, PTPRF was also reduced in EGI-1 cells upon miR-145-5p expression. Consistent with our finding that STAT1 and its target genes were not affected by miR-145-5p in HCC cells, PTPRF was not altered in the HCC cell lines HuH1 and Hep3B (Fig. S8). MiR-145-5p also reduced PTPRF at the protein level in biliary tract cancer cell lines (Fig. 5A,B).

Using multiple online tools to predict miRNA target gene binding, we were not able to identify canonical miR-145-5p binding sites in in the 3′UTRs of PTPRF or PTPRS, suggesting an indirect regulation of these genes by miR-145-5p. However, we identified two non-canonical miR-145-5p binding sites in the coding sequence (CDS) of PTPRF (Fig. 5C). Although microRNAs usually regulate gene transcripts upon binding to conserved 3′UTR binding sites, some studies support a functional role for coding region microRNA binding sites^26^. To further elucidate, whether these non-canonical miRNA-binding sites might contribute to direct regulation of PTPRF by miR-145-5p, both PTPRF CDS fragments spanning the potential miR-145-5p binding sites were inserted into pGL3-Promoter vector backbones. Subsequent luciferase reporter assay analysis revealed that both non-canonical miR-145-5p binding sites were insufficient to regulate PTPRF expression and that the downregulation of PTPRF and PTPRS is indirect (Fig. 5D). Summarizing, these data showed that PTPRF and PTPRS are downregulated most likely indirectly in miR-145-5p expressing BTC cell lines.

### The STAT1-target IRF7 is reduced in GBC and associated with poor clinical outcome

**Figure 6:**
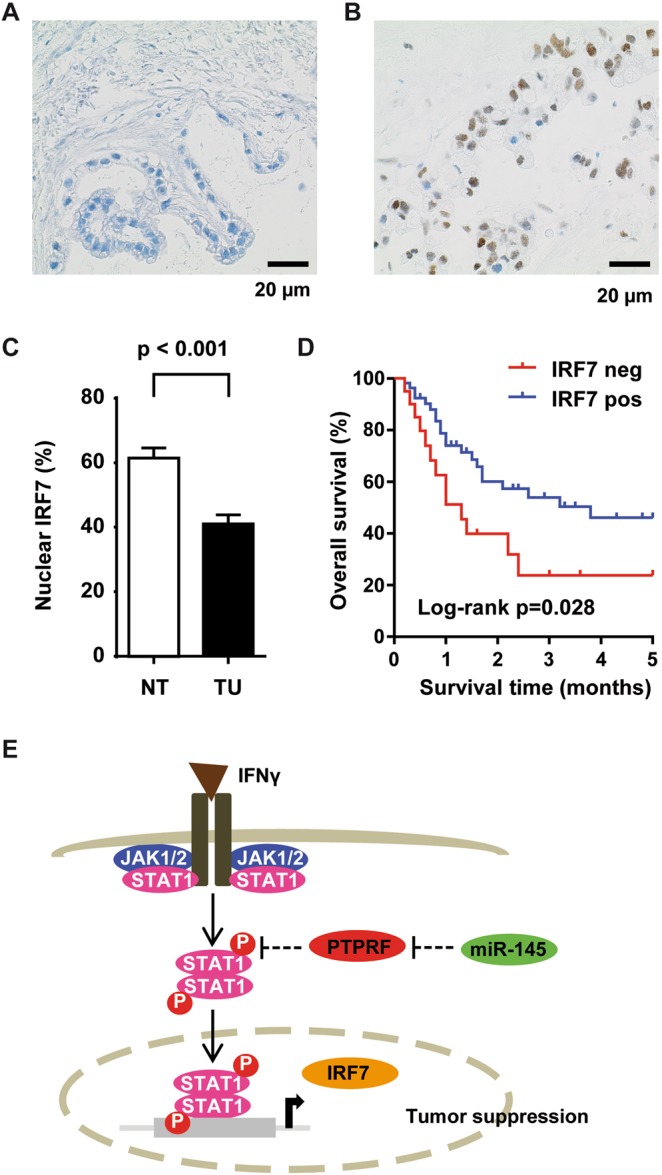
The IFNγ/STAT1 regulated gene IRF7 is down regulated and prognostic in GBC. (**A**) Immunohistochemical staining of IRF7 showed nuclear positivity. Immunohistochemical staining of one representative GBC case negative for IRF7 and **(B)** of one representative GBC case positive for IRF7, respectively. **(C)** Percentage of GBC tissues (N = 122) positive for nuclear IRF7 staining and of paired normal gallbladder tissue (N = 73). **(D)** Kaplan-Meier survival curves of GBC patients positive (N = 55) or negative (N = 20) for IRF7 immunohistochemical staining. Survival data was available for 75 out of 122 GBC patients. **(E)** Schematic model of miR-145-5p-mediated activation of STAT1 signaling. In BTC tumor cells, miR-145-5p expression leads to repression of PTPRF protein expression and increased STAT1 phosphorylation and transcriptional activity.

Since IRF7 has been demonstrated to be a direct target of STAT1 which directly bound to the IRF7 promoter and because PTPRS modulated IRF7 nuclear expression, we studied IRF7 expression in human GBC tissues^22,24,25,27^. We performed immunohistochemical staining of tissue microarrays and found that IRF7 mainly localizes to the nucleus in GBC and paired normal gallbladder epithelium (Fig. 6A,B). Consistently with the tumor suppressive role of STAT1 signaling in tumorigenesis, nuclear IRF7 protein expression was significantly reduced in GBC compared to normal gallbladder epithelium of paired samples (p < 0.001; Fig. 6C). Low IRF7 expression was significantly associated with poor outcome of GBC patients (Fig. 6D). In summary, the STAT1-target IRF7 and miR-145-5p are reduced in GBC patients with poor outcome suggesting that miR-145-5p is linked to STAT1 activation which we demonstrated in BTC cell lines. Thus, PTPRF and PTPRS are downregulated in miR-145-5p expressing biliary tract cell lines and may be involved in the release of STAT1 signaling blockade resulting in STAT1 target gene expression (Fig. 6E).

## Discussion

Few molecular studies exist on GBC biology and most studies focused on single molecules without presenting systematic screening analyses of a large cohort^17–19^. In this study, we performed miRNA profiling of a European cohort of 40 GBC patients with comprehensive clinical and follow-up data. Consistent with epidemiologic data, our cohort mainly consisted of older patients with a mean age of 67 years and advanced tumor stage disease^28^. UICC stage was mostly III or IV and in line with epidemiological survival data, median patient survival of our cohort was only 17.2 months^28,29^. As expected, we identified large differences between tumor and non-neoplastic gallbladder tissues reflecting major alterations in miRNA expression during tumorigenesis. Moreover, the integration of patient survival data allowed the identification of 24 differentially expressed miRNAs in patients with short versus long survival (Table S1). Interestingly, none of the clinical parameters was significantly different between long and short survival patient groups. Thus, this data set revealed multiple potentially important miRNAs and distinct GBC subgroups with differential miRNA expression. In addition, this data set is a valuable repository for future studies.

Functional assays in cell lines confirmed that miR-145-5p, which was the most intensely downregulated miRNA in tumor tissues of our study, exhibits a tumor suppressive function^19^. To identify key genes deregulated by miR-145-5p in biliary tract cancer cells, we performed whole genome gene expression profiling. Interestingly, most of the genes were upregulated upon miR-145-5p expression. Target gene activation by miRNAs has been reported to be mediated by multiple mechanisms. These include network-based mechanisms, involvement of enhancer and translational regulation^30–32^. In miR-145-5p expressing cells, the downstream pathway with the strongest deregulation was STAT1 signaling. Multiple direct and indirect STAT1 target genes have been activated upon miR-145-5p expression. Interestingly, we could confirm that STAT1 mRNA and protein were upregulated and STAT1 phosphorylation at tyrosine 701 and serine 727 was increased. In contrast, STAT1 was neither upregulated nor phosphorylated in HCC cells suggesting that this signaling mechanism present in biliary tract cancer cells displays cell type-specificity. These findings also illustrate the importance of examining liver tumors of hepatocellular, cholangiocellular or mixed origin separately.

STAT1 signaling activation has been shown in multiple tumor entities to be tumor suppressive and to be involved in therapy resistance mechanisms^33–37^. Thus, our findings are in line with the tumor suppressive function of miR-145-5p and the activation of tumor suppressive STAT1 signaling. STAT1 is mainly activated via IFNγ which leads to STAT1 receptor dimerization and STAT1 tyrosine 701 phosphorylation. The main sources of IFNγ are CD4+ and CD8+ T cells in the tumor microenvironment, respectively^38,39^. Employing our previously published data set^40^, we found that 69.5% (41 out of 59) of cases showed infiltration of CD4+ T cells and 96.6% (57 out of 59) of cases had infiltration of CD8+ T cells, respectively. Thus, CD4+ and CD8+ T cells frequently infiltrate GBC tumor tissues, may lead to IFNγ stimulation of tumor cells and thereby, to activation of tumor suppressive STAT1 signaling.

However, the activation of STAT1 upon miR-145-5p expression is most likely non-canonical as JAK, TYK, PIAS and SOCS proteins were unaltered. Furthermore, the Leukocyte Antigen Related (LAR) family protein tyrosine phosphatases PTPRF and PTPRS were downregulated at the mRNA and protein level upon miR-145-5p expression. Only few studies have so far described the function of PTPRF and PTPRS. Crosslinking of PTPRS on the human plasmacytoid dendritic cell line Gen2.2 delayed induction of p-STAT1 and inhibited nuclear translocation of the STAT1 target gene IRF7^24,25^. In this study, we found that IRF7 mainly localizes to the nucleus in GBC and normal gallbladder epithelium. Consistently with previous studies, IRF7 expression was significantly reduced in GBC compared to normal gallbladder epithelium of paired samples and low IRF7 expression was significantly associated with poor outcome (Fig. 6). Therefore, the STAT1-target IRF7 and miR-145-5p are reduced in GBC patients with poor outcome suggesting that miR-145-5p is linked to STAT1 activation which we have demonstrated in BTC cell lines.

PTPRF has been shown previously to inhibit β-catenin phosphorylation leading to reduced cell migration and inhibition of a rat bladder tumor cell line in a xenograft mouse model^41^. Another study in the human embryonic kidney cells HEK293 demonstrated that PTPRF dephosphorylates the death-associated protein kinase (DAPK) which has been suggested to be involved in colon cancer progression^42^. Interestingly, PTPRF is downregulated in a subset of HCC, gastric and colorectal cancer patients and has been suggested by Bera *et al*. to be involved in cell-cell contact-mediated ERK-signaling^43^. However, the function of PTPRF in HCC and BTC may be different as miR-145-5p did not induce STAT1 activation in HCC cell lines. The miRNA tools miRWalk and TargetScan predicted no canonical binding sites of miR-145-5p to PTPRF or PTPRS but two non-canonical sites in PTPRF were found. However, these two non-canonical miR-145-5p binding sites were not functional. This suggests that miR-145-5p acts indirectly or at the protein level. Although we were not able to show direct effects of miR-145-5p on PTPRF or PTPRS, our results suggest an important functional role of miR-145-5p in biliary tract cancer and clearly demonstrate the activation of STAT1 signaling by miR-145-4p.

Among the 31 by miR-145-5p most downregulated genes, three genes were predicted by TargetScan (www.targetscan.org/) to be direct miR-145-5p target genes (Table S3). These three genes were CMTM4, GNAS and C12orf49. C12orf49 is predicted to encode secreted protein which is also named UPF0454, however, nothing is known about the function of this protein. For CMTM4 only few publications exist to date. A recent study demonstrated a role for CMTM4 and its close family member CMTM6 in regulation of PD-L1^44^. Mezzadra *et al*. demonstrated that CMTM4 and CMTM6 directly interact with PD-L1 thereby stabilizing PD-L1 protein stability^44^. They suggest that CMTM6/4 may be exploited as therapeutic targets, either in isolation, or to enhance the effectiveness of the current PD-L1–PD-1 blocking therapies. Thus, our finding that CMTM4 is downregulated by miR-145-5p is in agreement with these findings. Moreover, GNAS may be a direct target of miR-145-5p. GNAS is a stimulatory G protein α subunit that is involved in classical activation of adenylyl cyclase in many cellular responses and activating mutations in GNAS at codon 201 encoding a p.Arg201Cys alteration have been identified in CCA^45^. Therefore, the potential downregulation of GNAS by miR-145-5p would be consistent with the here proposed tumor suppressive role of miR-145-5p.

MiR-145-5p has also been demonstrated to be tumor suppressive in HCC^46,47^. However, the mechanism of miR-145-5p action is likely not mediated through STAT1 signaling in HCC as we did not detect any differences in STAT1 signaling expression in HCC cell lines. Thus, miR-145-5p functions through multiple signaling pathways depending on the cell type which results to different mechanisms of tumor cell inhibition by miR-145-5p.

GBC treatment options are very limited today because most patients are diagnosed at unresectable, advanced tumor stages. Unfortunately, resection is the only potentially curative treatment option to date. The development of targeted therapies has been hampered by the low incidence of GBC and the lack of well characterized patient cohorts with available clinical data. Genomic sequencing studies demonstrated that GBC is a distinct tumor entity and mutational profiles differ greatly between GBC and other gastrointestinal tumor entities^10,11,48^. However, a recent review suggested that the mutational landscape of GBC may merit investigation of potentially actionable genomic alterations in GBC patients^49^. An example is HER2 amplification in GBC patients. Promising results have been observed in GBC patients with HER2 gene amplification or overexpression using HER2-targeted therapy which led to complete response or disease stability^50,51^. Therefore, it will be important to better understand the molecular mechanisms underlying GBC development and the underlying signaling mechanisms to improve overall patient survival.

Taken together, using a homogeneous German GBC patient cohort, we were able to identify distinct miRNA expression patterns in patients with poor outcome. Our systematic screening approach revealed a novel signaling mechanism of miR-145-5p inducing STAT1 signaling. Thus, these results may help developing new treatment modalities for this rare tumor entity.

## Materials and Methods

### Patients

FFPE tissue blocks of 8 normal gallbladders and 40 GBC tissues were included in this study. All tissue samples were provided by the Tissue Bank of the National Center for Tumor Diseases (NCT, Heidelberg, Germany) in accordance with the regulations of the Tissue Bank and with approval of the ethics committee of the University of Heidelberg. All analyses were carried out in accordance with the relevant guidelines and regulations of University of Heidelberg and informed consent was obtained from all participants. All 40 GBC patients underwent resection at Heidelberg University Hospital at the time of diagnosis and did not receive any treatment prior sample collection. Each GBC case was histologically confirmed by at least two specialized pathologists of the Institute of Pathology, Heidelberg University Hospital. Non-neoplastic gallbladder tissues without severe inflammation were obtained from patients who underwent cholecystectomy due to gallstone disease and were used in this study as normal tissue reference.

### Preparation of tissue microarrays and immunohistochemistry

In this study GBC tumor tissue microarrays and tissue microarrays of normal non-neoplastic gallbladder tissue of the same GBC cohort were generated. Tissue cores of 1.0 mm diameter were extracted from the donor blocks and embedded into a new paraffin array block using a tissue microarrayer (TMA Grand Master Fa. Sysmex, Germany). For the invasive GBC tumor, a duplicate TMA was punched out from a separate area, respectively, to increase study sensitivity and specificity.

Immunohistochemistry was performed on an automated immunostainer (Ventana BenchMark ultra, Roche Diagnostics, Rotkreuz, Switzerland) using the biotin-free OptiView DAB IHC Detection Kit (Roche Diagnostics). In brief, the formalin fixed and paraffin-embedded TMA blocks were cut into 3 µm thin sections, deparaffinized and rehydrated. Heat-induced epitope retrieval was performed using Ultra CC1 (Cell Conditions Solution). After blocking of endogenous peroxidase, the slides were incubated with the rabbit anti-IRF7 antibody (HPA052757/Atlas Antibodies, Bromma, Sweden) at a dilution of 1:100 followed by incubation with OptiView Universal Linker and OptiView HRP Multimer. Visualization was achieved using DAB-Chromogen. Before mounting, slides were counterstained with hematoxylin.

### Cell lines

Two hepatocellular carcinoma cell lines (HuH1 and Hep3B), the iCCA cell line HuCCT1, two biliary tract cancer cell lines (EGI-1 and TFK-1), two GBC cell lines (OZ and Mz-ChA-1) and HEK293T cells were used in this study. Cell lines were obtained from ATCC (Hep3B and HEK293T), DSMZ (EGI-1 and TFK-1) or JCRB (HuH1, HuCCT1 and OZ). Mz-ChA-1 cells were a gift from Xin Chen (UCSF, San Francisco, USA).

HuH1, EGI-1, Mz-ChA-1 and HEK293T cells were cultured in Dulbecco’s Modified Eagle’s Medium (DMEM) and Hep3B cells in Minimum Essential Medium (MEM). HuCCT1 and TFK1 were maintained in RPMI-1640 medium and OZ cells in William’s E Medium. All media were supplemented with 10% fetal calf serum and 1% Penicillin-streptomycin (100 IU/mL and 100 g/mL, respectively). All media and supplements were obtained from Sigma-Aldrich (St. Louis, MO, USA) and all cell lines were incubated at 37 °C with 5% CO_2_. Cell lines were transfected using RNAiMAX for miRNA mimics or AllStars control. Lipofectamine 2000 transfection reagent was used for plasmid DNA transfection (Life Technologies Darmstadt, Germany) according to the manufacturer’s instructions.

### RNA isolation

For RNA isolation of normal gallbladder tissues and human GBC tumor samples, tumor tissue areas were selected based on hematoxylin and eosin staining of formalin-fixed and paraffin-embedded (FFPE) tissue blocks. MiRNA samples for microarray hybridization were purified from microdissected FFPE material using the miRNeasy FFPE Kit (Qiagen, Hilden, Germany), according to the manufacturer’s instructions. Microdissection was performed as previously described^52^. Subsequently, FFPE material was deparaffinized followed by lysis with Proteinase K and DNase digestion. The extracted RNA including miRNA was quantified with the Nanodrop ND-1000 spectrophotometer (NanoDrop Technologies, Rockland, Germany) and the Agilent 2100 Bioanalyzer (Agilent Technologies, Palo Alto, CA). Total RNA of cell lines was isolated using TRIzol Reagent (Invitrogen, Life Technologies, Carlsbad, CA) according to the manufacturer’s instructions and quantified with the Nanodrop ND-1000 spectrophotometer and the Agilent 2100 Bioanalyzer (Agilent Technologies, Palo Alto, CA).

### MiRNA microarrays and data analysis

For miRNA profiling of normal gallbladder tissues and human GBC tumor samples, Agilent SurePrint Human miRNA Microarray (G4872A, miRBase Release 19.0, Agilent Technologies, Santa Clara, CA) representing 2006 human miRNAs were used. Labelling, hybridization and processing of the data were done according to the manufacturer’s recommendations. In total, 200 ng of RNA was labelled and hybridized to each array using standard Agilent protocols. Microarrays were scanned on an Agilent Technologies Scanner G2505C and signal intensities were preprocessed and normalized by the Agilent feature extraction software. The miRNA data analysis was performed using R package limma available at Bioconductor (www.bioconductor.org). The algorithms were implemented in the R programming language (www.r-project.org). MiRNA expression levels of the 8 non-neoplastic gallbladder tissues and 40 GBC tumor samples were normalized together using quantile normalization and were subsequently log2 transformed. Differential expression of miRNAs was analyzed using limma’s T-test class comparison for the respective groups, as indicated. All miRNA expression data have been deposited into the Gene Expression Omnibus repository (GEO, http://www.ncbi.nlm.nih.gov/geo/), with serial accession number GSE104165.

### Gene expression microarrays and data analysis

Gene expression profiling was performed using Human Gene 1.0 ST Arrays from Affymetrix (High Wycombe, UK). Biotin-labelled complementary RNA was prepared according to the Affymetrix standard labelling protocol. Complementary RNA was purified, fragmented and hybridized using a GeneChip Hybridization oven 640, stained using a GeneChip Fluidics Station 450 and scanned with a GeneChip Scanner 3000 (Affymetrix). A Custom CDF Version 20 with ENTREZ based gene definitions was used to annotate the arrays^53^. Raw fluorescence intensity values were normalized by quantile normalization and differential gene expression was calculated by ANOVA using the software package SAS JMP7 Genomics (SAS Institute, Cary, NC). A false positive rate of α = 0.05 with FDR correction was taken as the level of significance. Gene Set Enrichment Analysis (GSEA) was used to determine whether defined lists (or sets) of genes exhibit a statistically significant bias in their distribution within a ranked gene list using the software GSEA^54^. The Pathways were obtained from public external databases (KEGG, http://www.genome.jp/kegg). Raw and normalized data were deposited into GEO (http://www.ncbi.nlm.nih.gov/geo/), with serial accession number GSE102815.

### Pathway analysis and statistics

Genes deregulated in TFK-1 cells transfected by miR-145-5p compared to AllStars control were analyzed by Ingenuity Pathway Analysis (IPA; http://www.ingenuity.com/products/ipa). We performed upstream IPA analysis of the genes with adjusted p < 0.1 and log expression difference of at least 0.3 which resulted in 121 genes. The log expression ratios were used to predict upstream regulators. The mechanistic networks of upstream regulators with the highest activation scores were further analyzed.

The statistical significance was defined as p < 0.05. Correction for multiple testing was performed by Benjamini–Hochberg or FDR adjustment, as indicated. Fisher’s exact test for more than two groups was performed using the stats package in R.

Additional materials and methods are included in the Supplemental Data.

### Ethics approval

Human samples were provided by the tissue bank of the National Center for Tumor Diseases (NCT, Heidelberg, Germany) in accordance with the regulations of the tissue bank and the approval of the ethics committee of Heidelberg University.

## Supplementary information

Supplemental Material
