# Supplementary material for: Profiling of gallbladder carcinoma reveals distinct miRNA profiles and activation of STAT1 by the tumor suppressive miRNA-145-5p
Source: Sci Rep. 2019 Mar 18;9:4796. doi: 10.1038/s41598-019-40857-3 (PMC6423323; doi:10.1038/s41598-019-40857-3)
Supplement: Supplementary file 1 — Supplemental Material [file 41598_2019_40857_MOESM1_ESM.docx]

***Supplemental data***

**Profiling of gallbladder carcinoma reveals distinct miRNA profiles and activation of STAT1 by the tumor suppressive miRNA-145-5p**

Benjamin Goeppert^1^, Felicia Truckenmueller^1^, Alessandro Ori^2^, Valerie Fritz^3^, Thomas Albrecht^1^, Angelika Fraas^1^, Dominique Scherer^4^, Rosa González Silos^4^, Carsten Sticht^5^, Norbert Gretz^5^, Arianeb Mehrabi^6^, Melanie Bewerunge-Hudler^7^, Stefan Pusch^8^, Justo Lorenzo Bermejo^4^, Peter Dietrich^3,9^, Peter Schirmacher^1^, Marcus Renner^1^, Stephanie Roessler^1^

**Supplemental Materials and Methods**

***Reverse Transcription and qRT-PCR***

For miRNA analysis, reverse transcription (RT) of 250 ng total RNA per sample was performed with the miScript RT Kit (Qiagen) according to the manufacturer's instructions. The qRT-PCR was carried out using respective miScript Primer Assays and miScript SYBR Green PCR Kit (Qiagen) on a StepOnePlus real-time PCR instrument (Applied Biosystems, Darmstadt, Germany). All samples were run in triplicates and relative quantification was carried out by using the comparative Delta Delta Ct (ΔΔCt) method and SNORD48 as an endogenous control.

For mRNA gene expression analysis, 1 µg total RNA was reverse transcribed by RevertAid H Minus First Strand cDNA Synthesis Kit (Thermo Fisher Scientific, Waltham, MA) and PCR reaction performed in triplicates using GoTaq qPCR Master Mix (Promega, Mannheim, Germany) on a StepOnePlus real-time PCR instrument (Applied Biosystems, Darmstadt, Germany). The reference gene serine/arginine-rich splicing factor 4 (SRSF4) was used as an internal control. Relative mRNA expression values were calculated using the comparative ΔΔCt method.

All miScript Primer Assays and qRT-PCR primer sequences are listed in Table S5.

***Western blot***

Total protein was extracted from cell lines with RIPA buffer (50 mM Tris-HCl pH 7.4, 150 mM NaCl, 1% Triton X-100, 1% sodium deoxycholate, 0.1% SDS, 1 mM EDTA pH 8.0) supplemented with PhosStop and protease inhibitor Complete Mini EDTA-free (Roche Diagnostics, Mannheim, Germany). The protein concentration of each sample was determined by Bradford assay (Sigma-Aldrich, Taufkirchen, Germany). Twenty µg of protein lysates were separated on 8% or 10% Bis/Tris-polyacrylamide gels and then transferred to an equilibrated nitrocellulose membrane (Amersham Biosciences, Buckinghamshire, UK). Membranes were blocked with 5% milk in TBST and immunoblotted with the indicated antibodies overnight at 4°C. Proteins were detected with IRDye secondary antibodies using an Odyssey Sa Infrared Imaging System (LI-COR Biosciences, Bad Homburg, Germany). Protein abundance was quantified using Image Studio v3.1.4 (LI-COR Biosciences). All primary and secondary antibodies are listed in Table S6.

***Cell viability assay***

TFK-1 or EGI-1 cells were seeded in 6 cm dishes and transiently transfected the next day. After 48 h, 2000 TFK-1 cells or 3000 EGI-1 cells were seeded in a 96-well plate in sextuplicates and viability was measured with CellTiter-Blue Cell Viability Assay (Promega, Mannheim, Germany). Twelve microliters of reaction solution diluted 1:2 in PBS and 60 µl medium was added to the cells, incubated for 1 h at 37°C, and measured with an Omega FLUOstar Microplate Reader (560Ex/590Em; BMG LABTECH, Ortenberg, Germany).

***Colony formation assay***

Forty-eight h after transfection, 4000 TFK-1 cells were seeded on a 6-well plate and cultured for 10–14 days. Then, cells were washed twice with PBS and stained with 0.5% crystal violet solution in 25% methanol for 20-30 min at room temperature. All experiments were conducted at least three times in triplicates.

***Dual-Luciferase Reporter Assay***

TFK-1 or EGI-1 cells were transfected with AllStars control or miR-145-5p mimic and 24 h later with firefly luciferase reporter vector pGL4 [luc2P/GAS-RE/Hygro] and pRL-TK (both Promega) using Lipofectamine 2000 transfection reagent. After additional 48 h, cells were either stimulated with 500 U/mL IFNγ (Sigma-Aldrich) for 8 h or left untreated. Luciferase activity was analysed by the Dual-Luciferase Reporter Assay System (Promega) according to the manufacturer’s protocol using an Omega FLUOstar Microplate Reader.

The PTPRF CDS fragment (114 bp) spanning the first potential miR-145-5p binding site was inserted into a pGL3-Promoter vector backbone. The XbaI sites used for insertion of the amplified cDNA fragment have remained intact. Likewise, the PTPRF CDS fragment (262 bp) spanning the second potential miR-145-5p binding site was inserted into a pGL3-Promoter vector backbone. The following primers were used: hPTPRF_3734for_CAG-XbaI: 5’-CAG TCT AGA CTC GCT TGT CAG GTG GTT CT-3’ and hPTPRF_4084rev_CAG-XbaI: 5’-CAG TCT AGA CTG GAC CAC GAT CTC ATC CG-3’. The renilla luciferase reporter plasmids containing the non-canonical CDS binding sites of the PTPRF gene (RLuc) or the firefly luciferase control plasmid pGL3 (Promega) (FLuc) were transfected into liver cells (PLC) which were used as a model system to detect miR-target gene interactions. All RLuc signals were normalized to the respective FLuc signals as ratio (RLuc/FLuc). Cells were co-transfected with either miR-145-5p or the AllStars-control microRNA together with the reporter plasmid RLuc or the control plasmid FLuc for normalization using Lipofectamine 2000 (Invitrogen) according to manufacturer's instructions. Luciferase assays were performed in 96-well plates at 48h post transfection.

**Supplemental Figures**

**
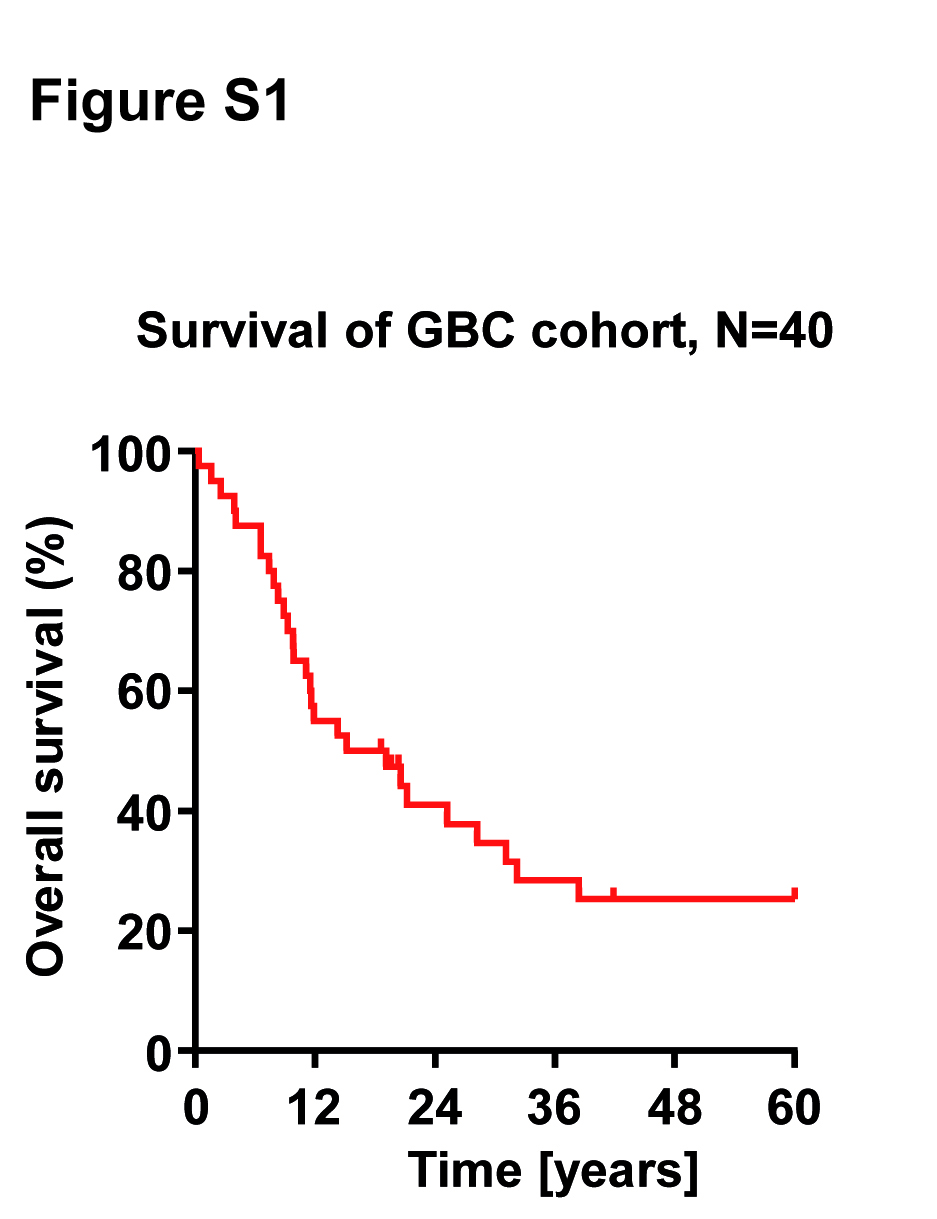
**

**Figure S1: Overall survival of GBC patient cohort.** Shown is the Kaplan-Meier overall survival curve of all 40 GBC patients used in this study. Survival data of all 40 GBC patients was available.

**
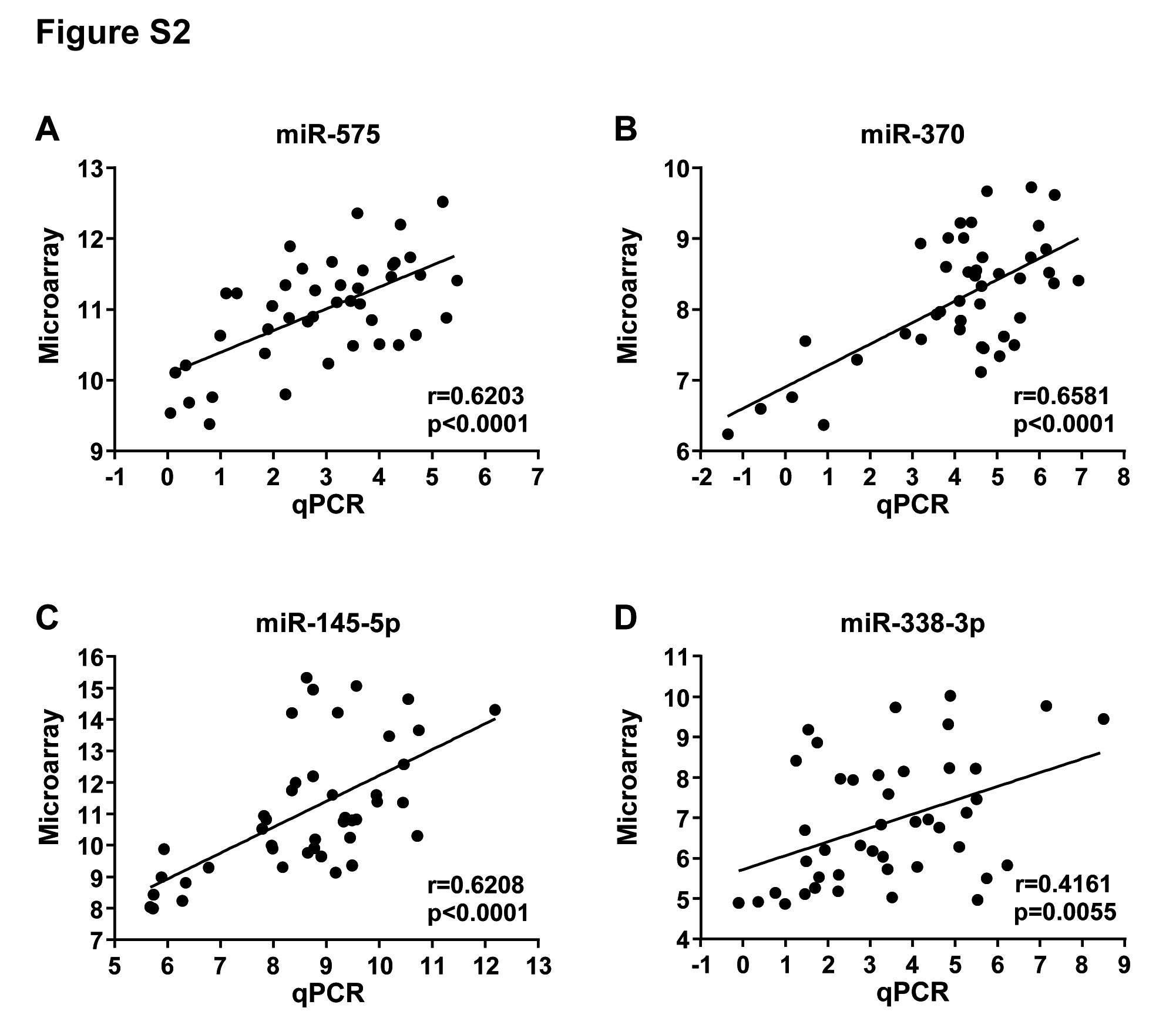
**

**Figure S2: Validation of microarray data.** Quantitative real time RT-PCR of miR-575 **(A)**, miR-370 **(B)**, miR-145-5p **(C)** and miR-338-3p **(D)** expression in the 8 normal gallbladder tissues and 35 GBC samples. 5 GBC samples had to be excluded due to low RNA amount.

**
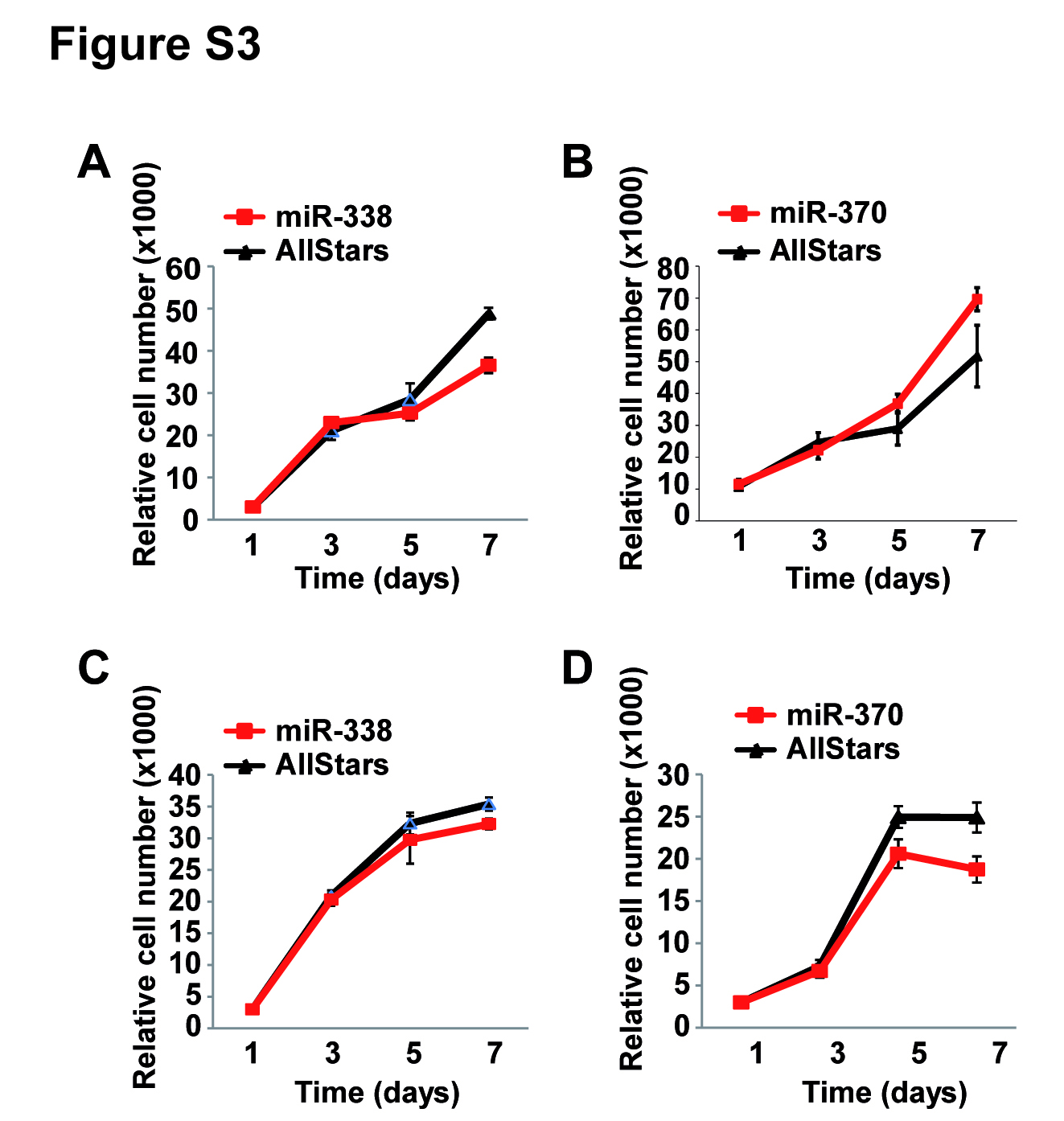
**

**Figure S3: Cell viability is not significantly altered upon miR-338-3p or miR-370 expression.** **(A and C)** Expression of miR-338-3p **(B and D)** or of miR-370 in EGI-1 or TFK-1 cells does not alter cell viability.

**
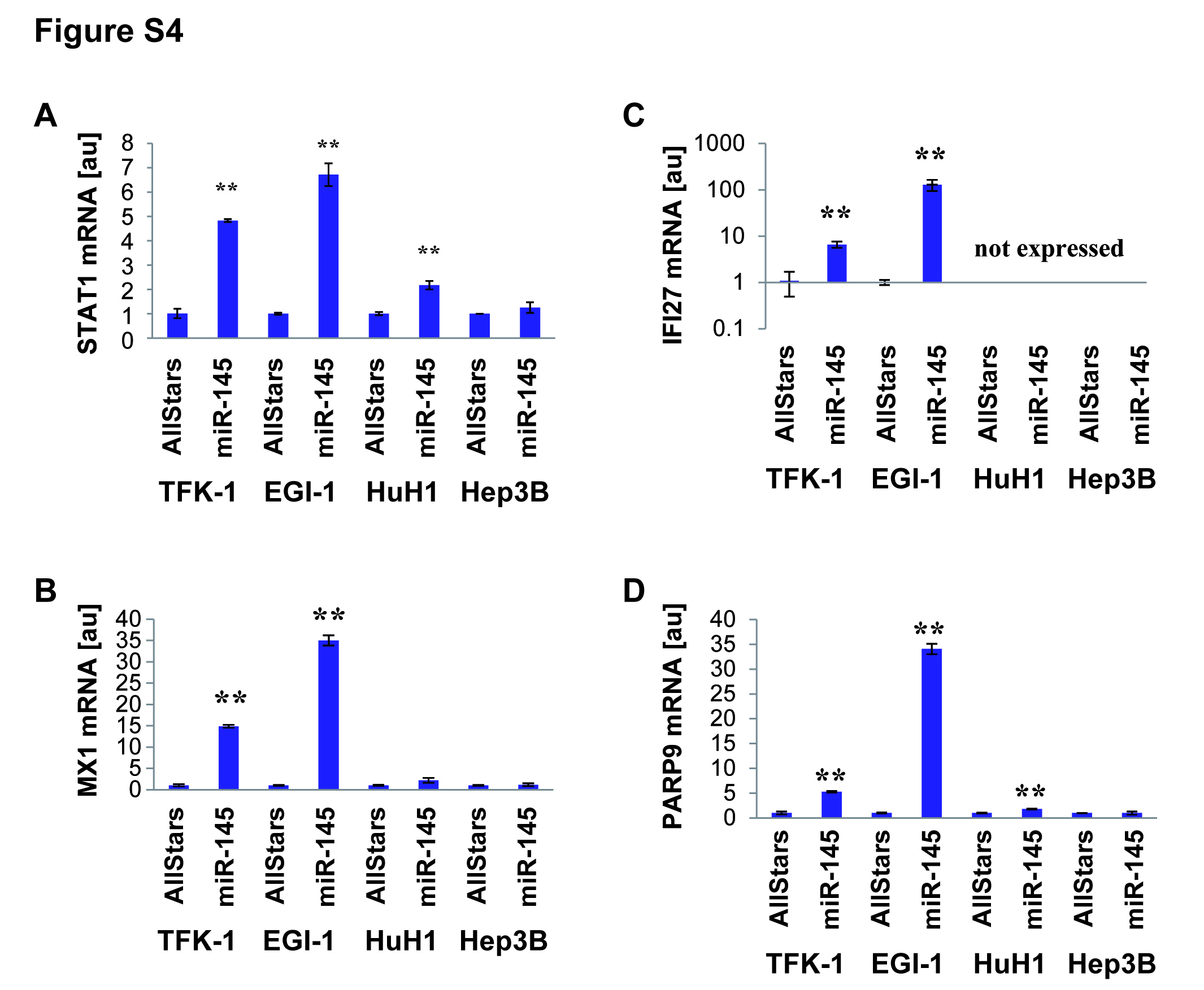
**

**Figure S4: MiR-145-5p expression induces the expression of STAT1 and its target genes IFI27, MX1 and PARP9 in CCA cell lines. (A)** Quantitative real time RT-PCR of STAT1, **(B)** IFI27, **(C)** MX1 and **(D)** PARP9 mRNA expression in the CCA cell lines TFK-1 and EGI-1 and in the HCC cell lines HuH1 and Hep3B cells transfected with AllStars control or miR-145-5p mimic. CCA: colangiocellular carcinoma; HCC: hepatocellular carcinoma.

**
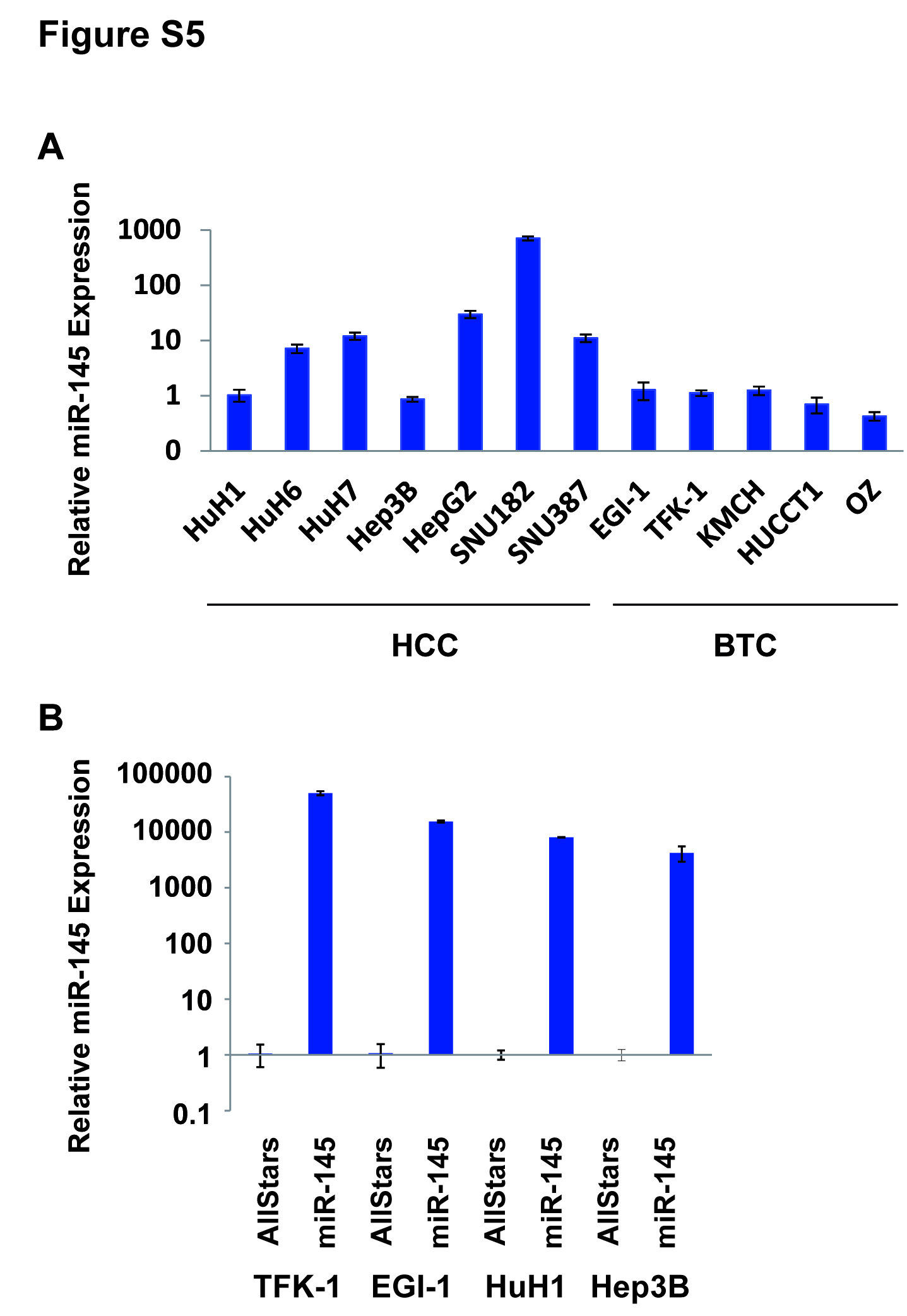
**

**Figure S5: Endogenous miR-145-5p expression and overexpression of miR-145-5p mimic. (A)** MiR-145-5p expression varies up to 1000-fold in cell lines and **(B)** over expression of miR-145-5p was very effective in CCA and HCC cell lines. BTC: biliary tract carcinoma; HCC: hepatocellular carcinoma.

**
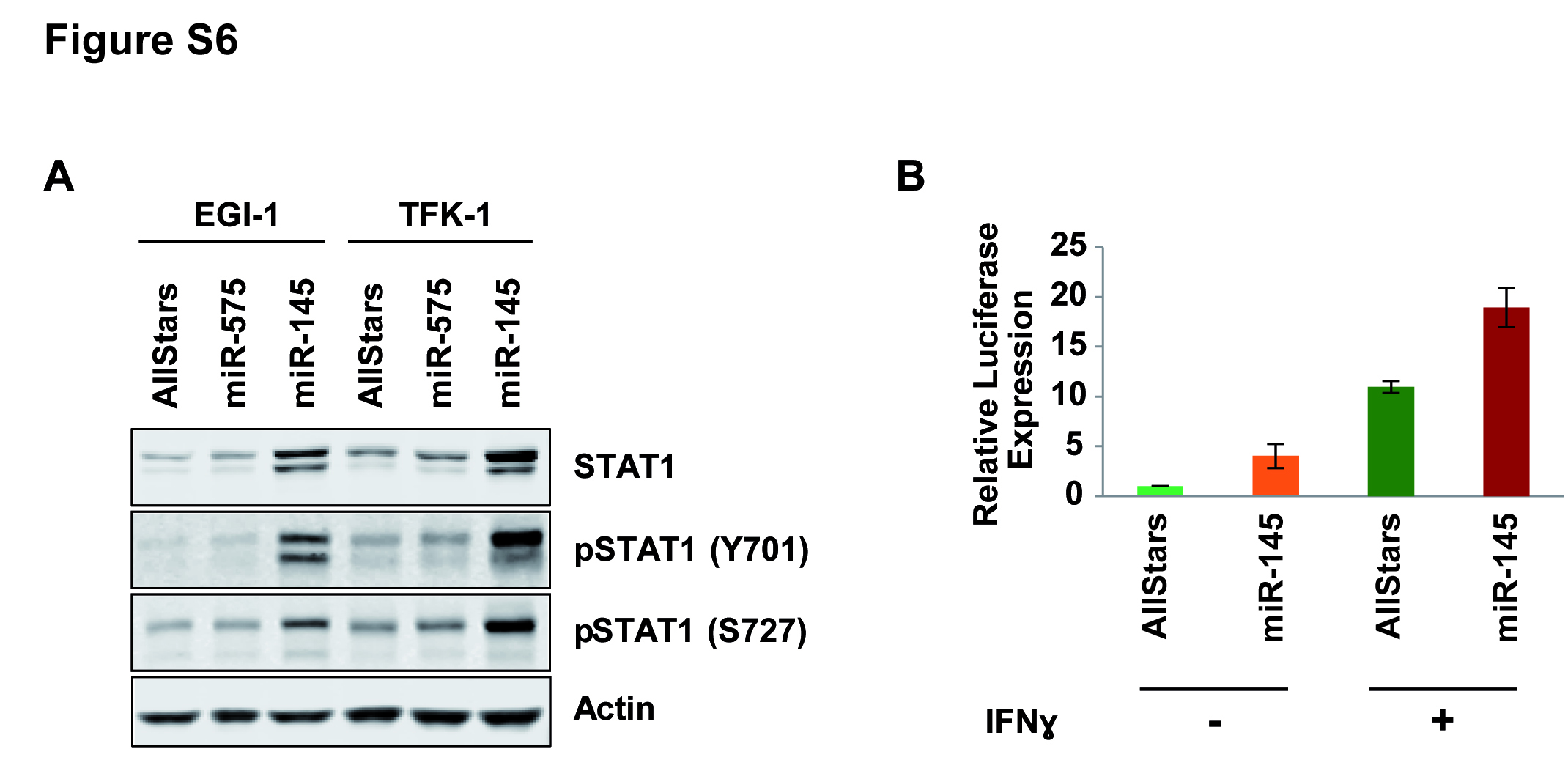
**

**Figure S6: Activation of STAT1 transcriptional activity.** **(A)** Western blot analysis of total STAT1, STAT1 phosphorylated at tyrosine 701 (pSTAT1-Y701) or STAT1 phosphorylated at serine 727 (pSTAT1-S727) in EGI-1 and TFK-1 cell lines transfected with control, miR-575 or miR-145-5p mimic, as indicated. Actin served as loading control. **(B)** Luciferase assay analysing STAT1 transcriptional activity using a GAS-luciferase reporter construct in EGI-1 cells expressing AllStars control or miR-145-5p. Prior harvesting cells were stimulated with or without 500 U/ml IFNγ for 8 h. Data represent mean ± SD of four independent biological experiments normalized to unstimulated AllStars control.

**
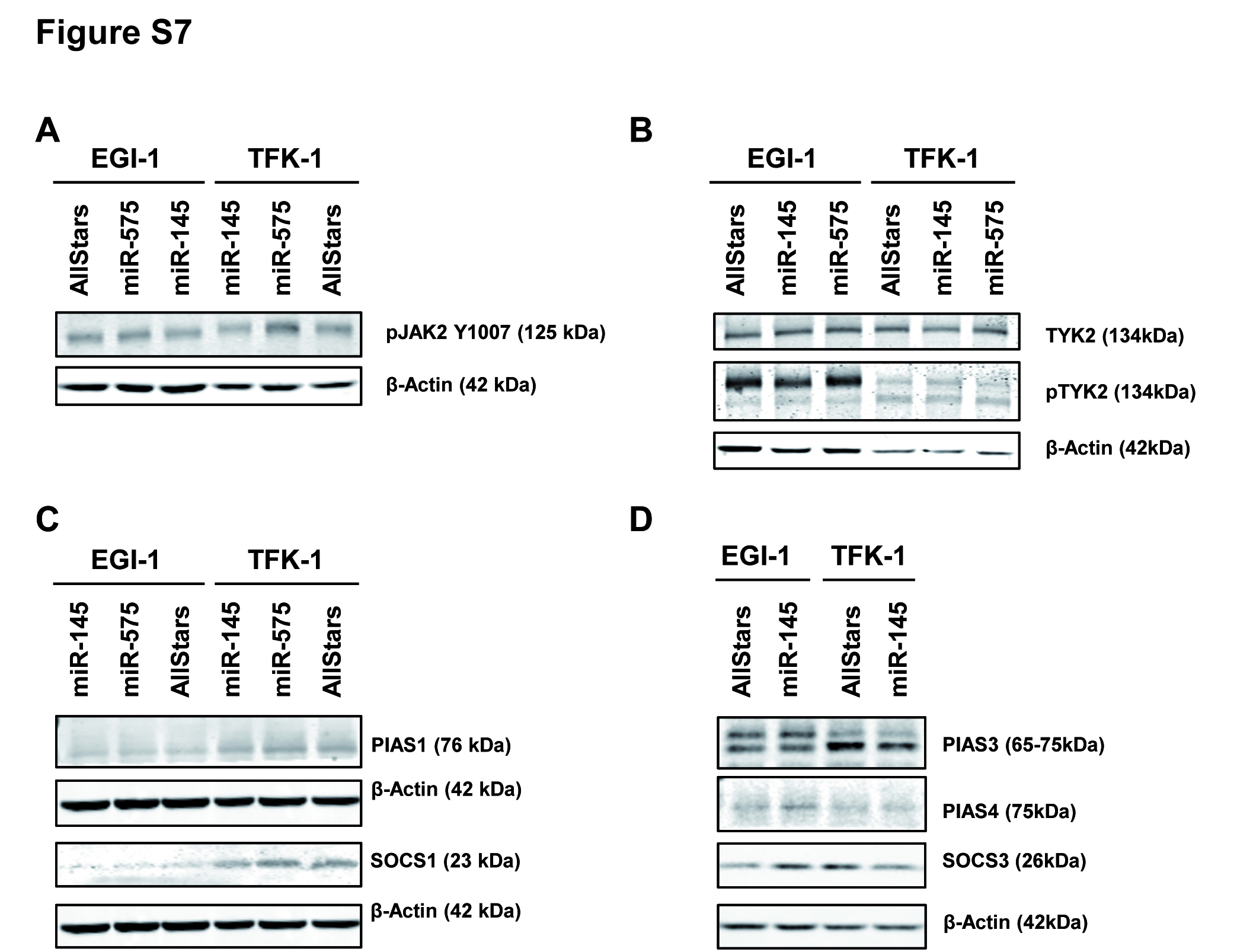
**

**Figure S7: Expression of STAT1 regulatory proteins upon miR-145-5p transfection. (A)** Western blot analysis of phosphorylated JAK2, **(B)** total or phosphorylated TYK2, **(C)** PIAS1, SOCS1, **(D)** PIAS3, PIAS4 and SOCS3 in EGI-1 and TFK-1 cell lines transfected with control, miR-575 or miR-145-5p mimic, as indicated. Actin served as loading control.

**
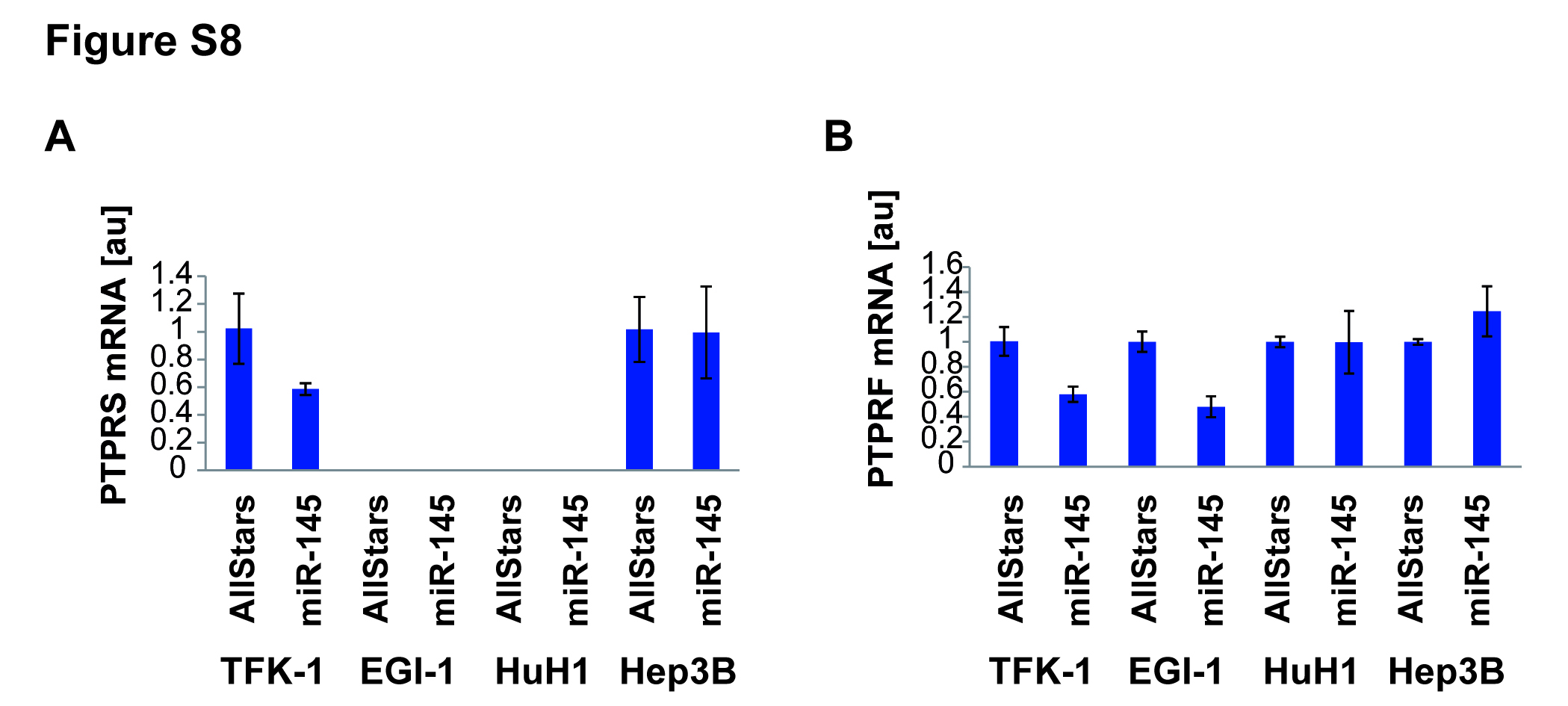
**

**Figure S8: Expression of PTPRS and PTPRF mRNA levels in CCA and HCC cell lines. (A)** Quantitative real time RT-PCR of PTPRS and **(B)** PTPRF mRNA expression in TFK-1, EGI-1, HuH1 and Hep3B cells transfected with AllStars control or miR145 mimic.

**Full-length images of Western blots**

**To Figure 4A-1**

**
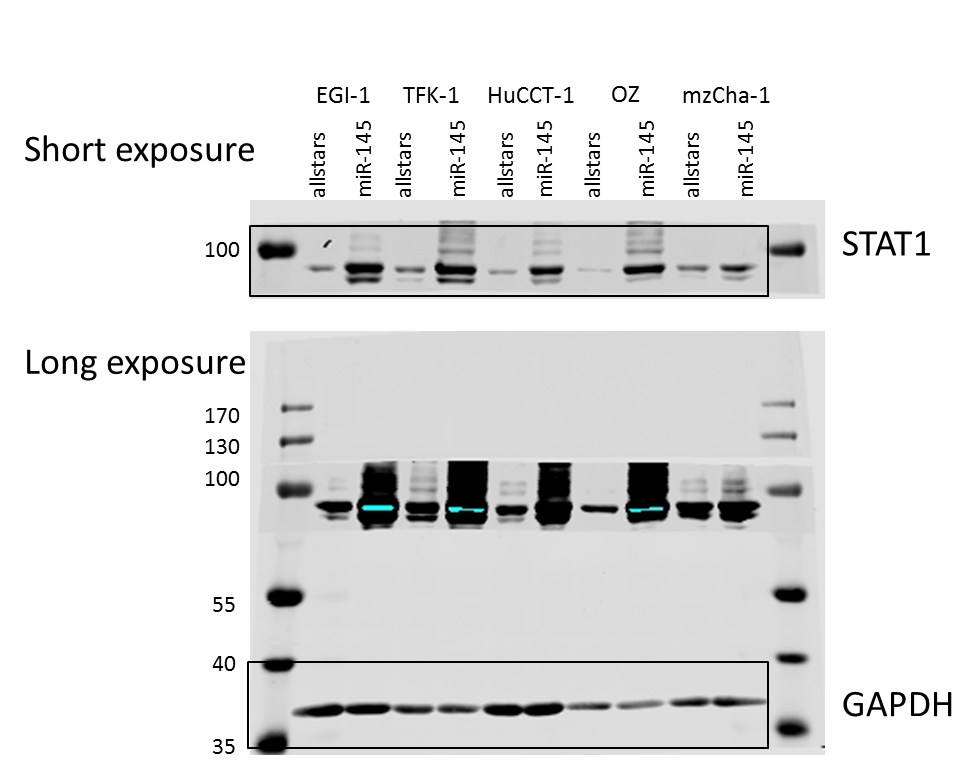
**

**To Figure 4A-2**

**
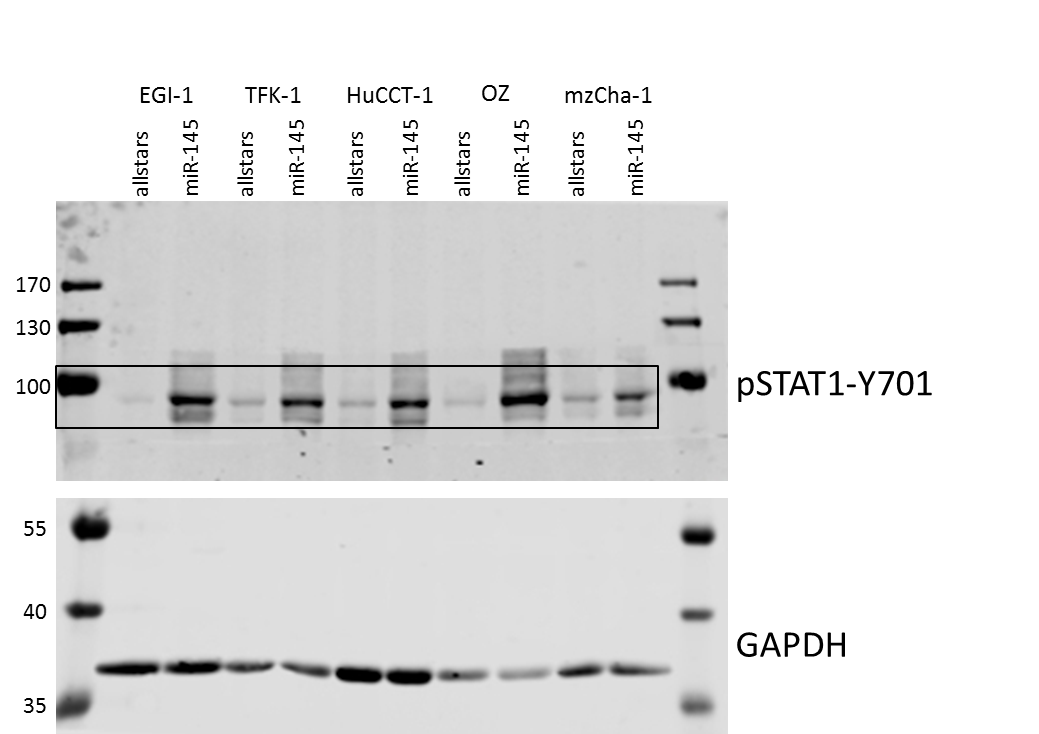
**

**To Figure 4A-3**

**
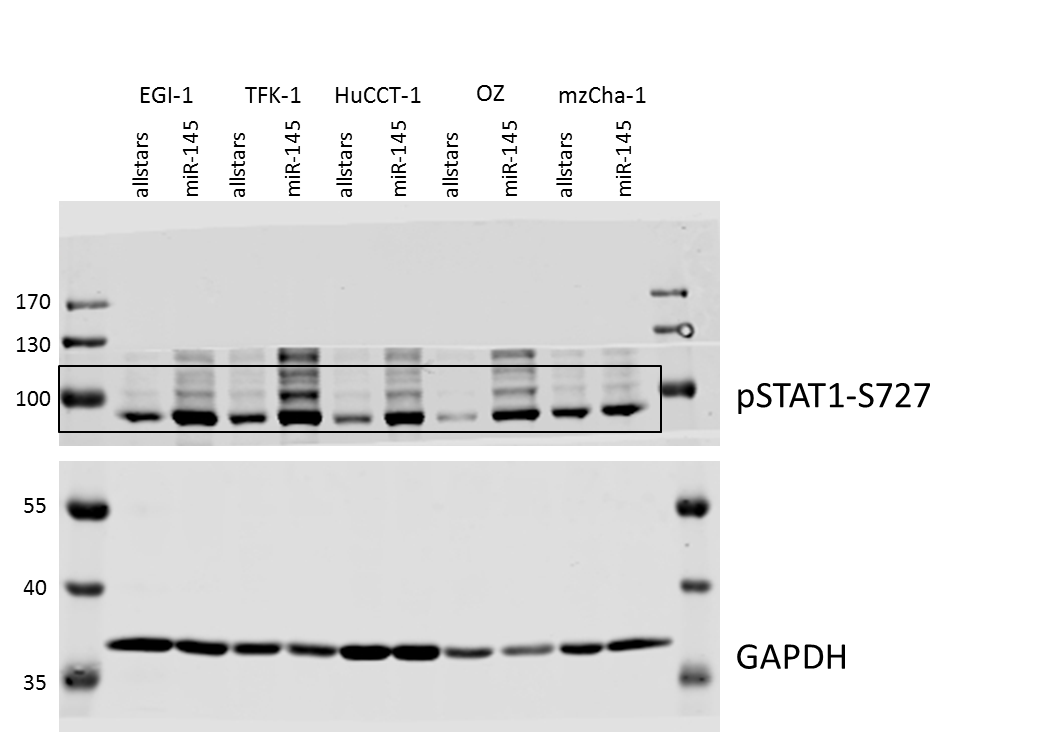
**

**To Figure 4C-1**

**
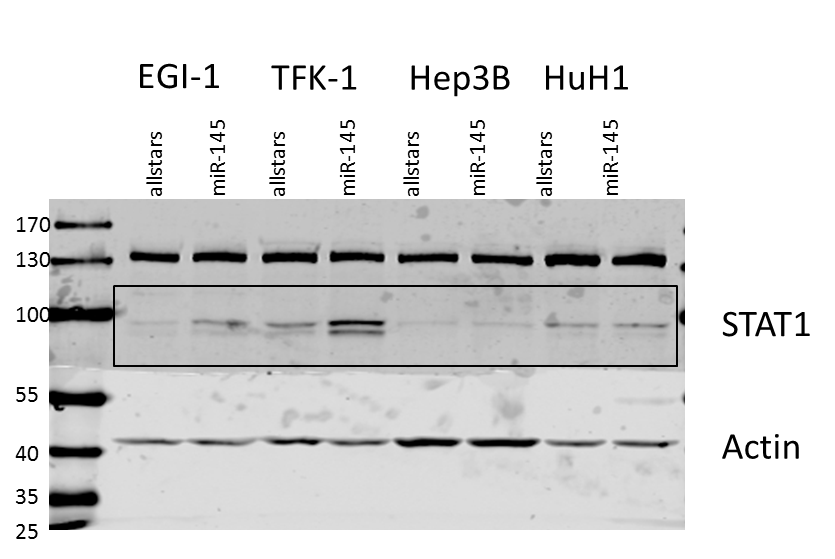
**

**To Figure 4C-2**

**
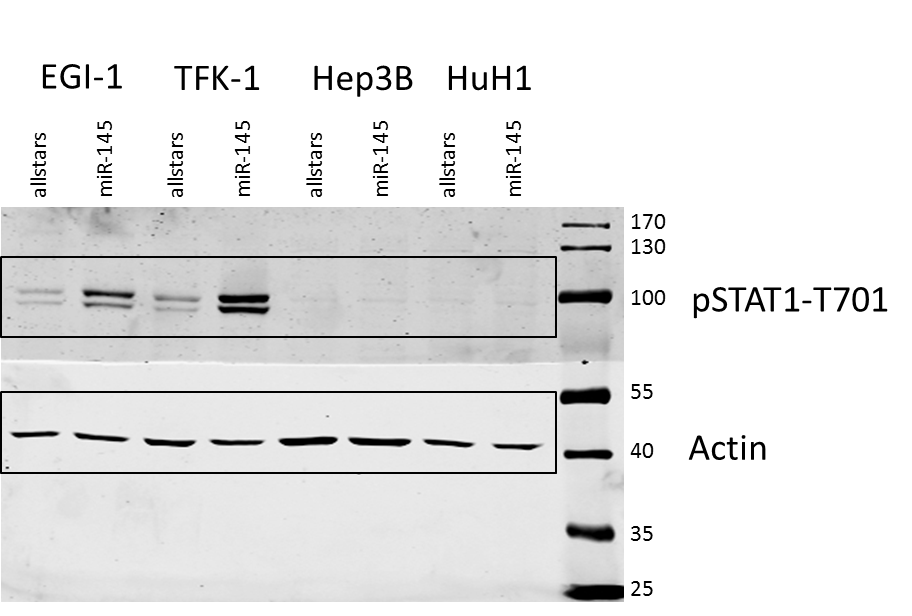
**

**To Figure 4C-3**

**
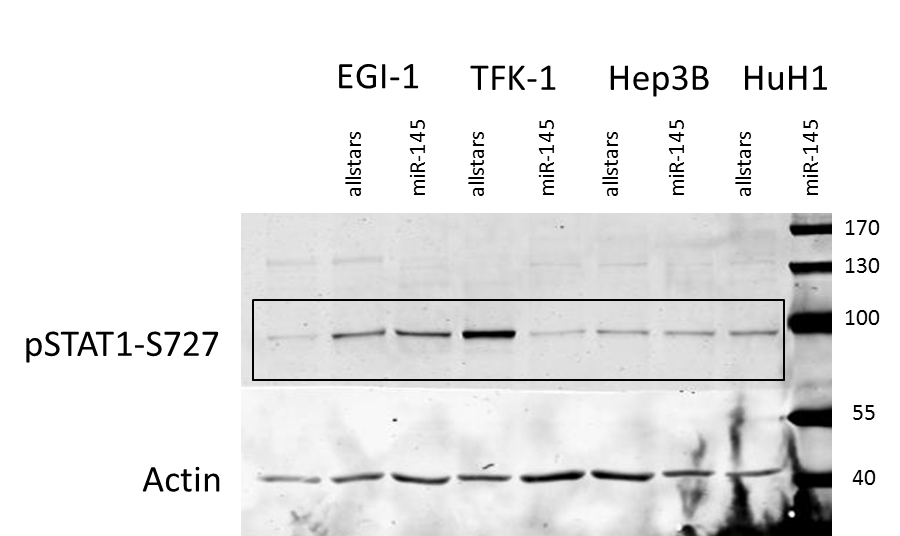
**

**To Figure 5**

**
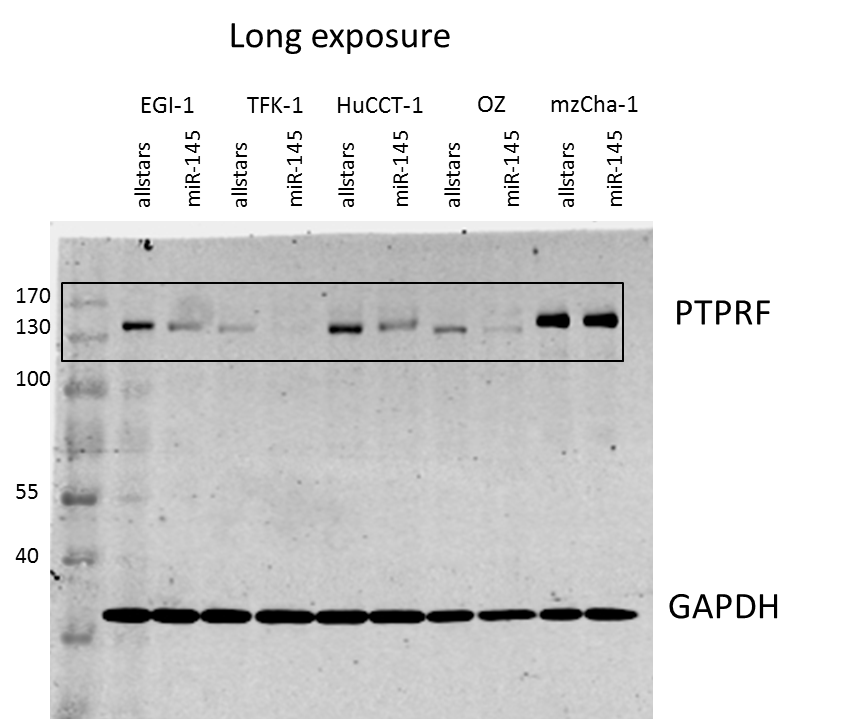
**

**
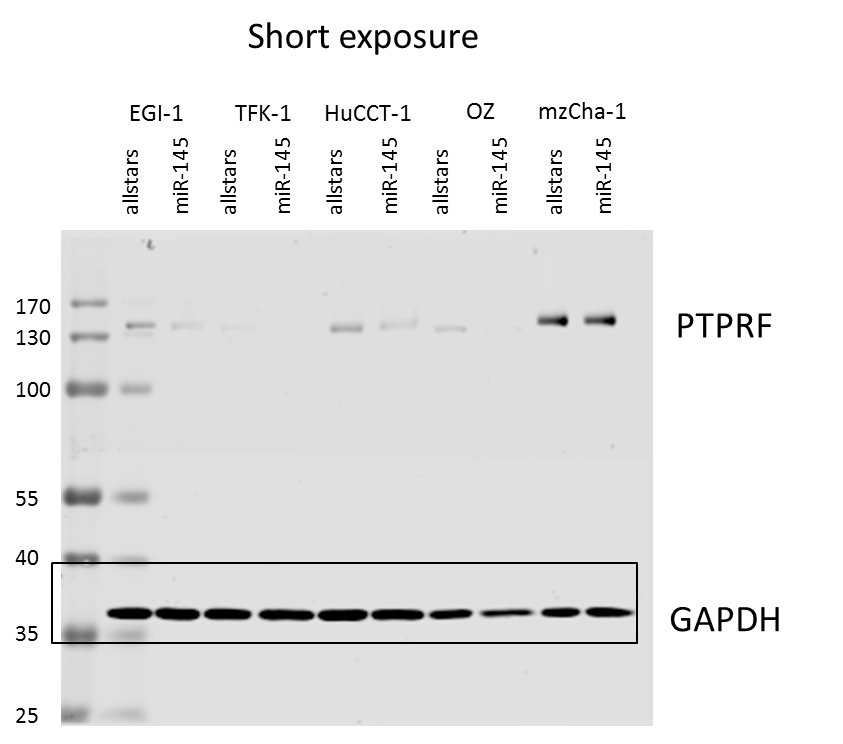
**

**Supplemental Figure S6A-1**

**
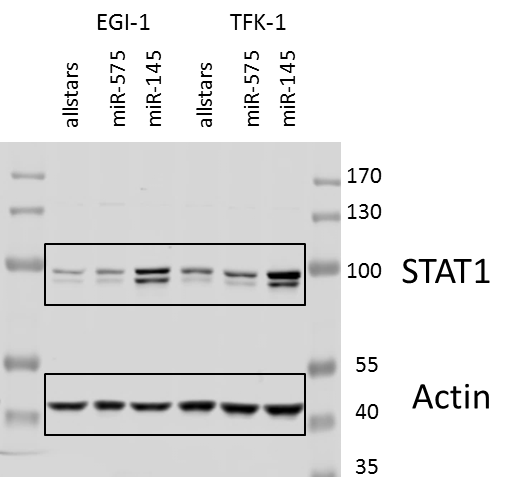
**

**Supplemental Figure S6A-2**

**
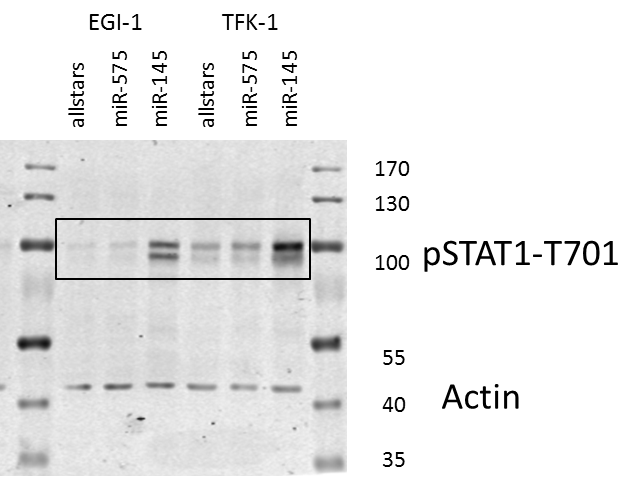
**

**Supplemental Figure S6A-3**

**
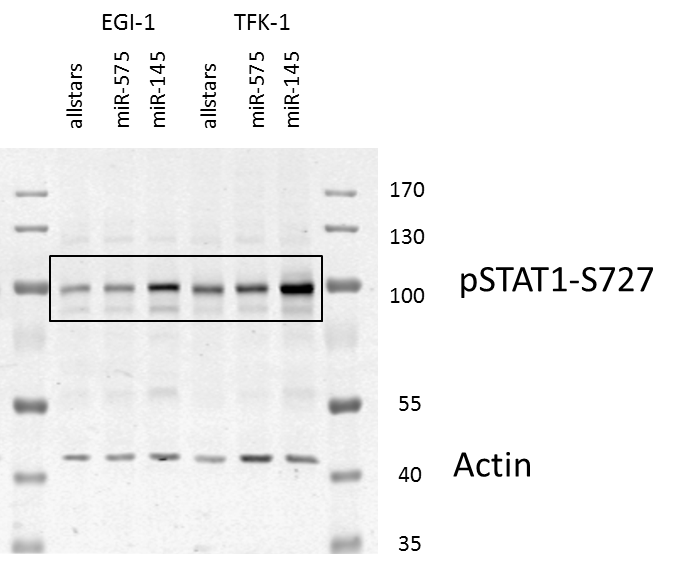
**

**Supplemental Figure S7A**

**
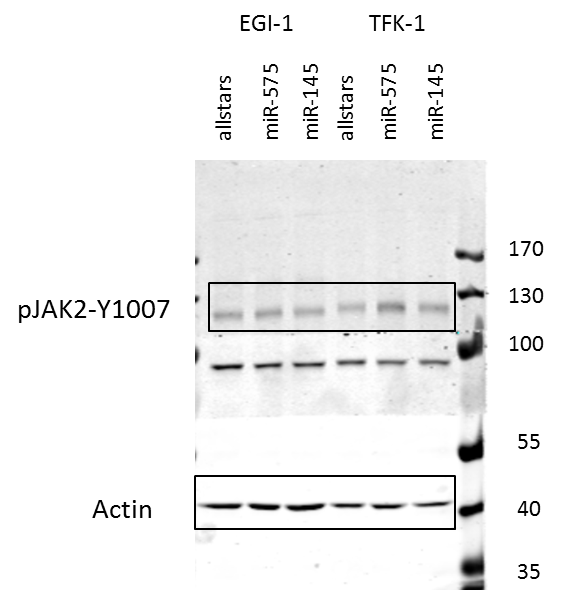
**

**Supplemental Figure S7B**

**
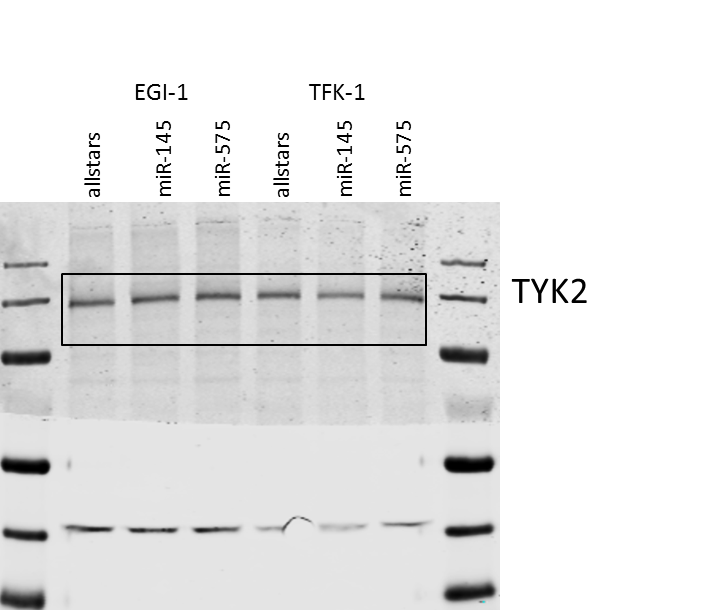
**

**
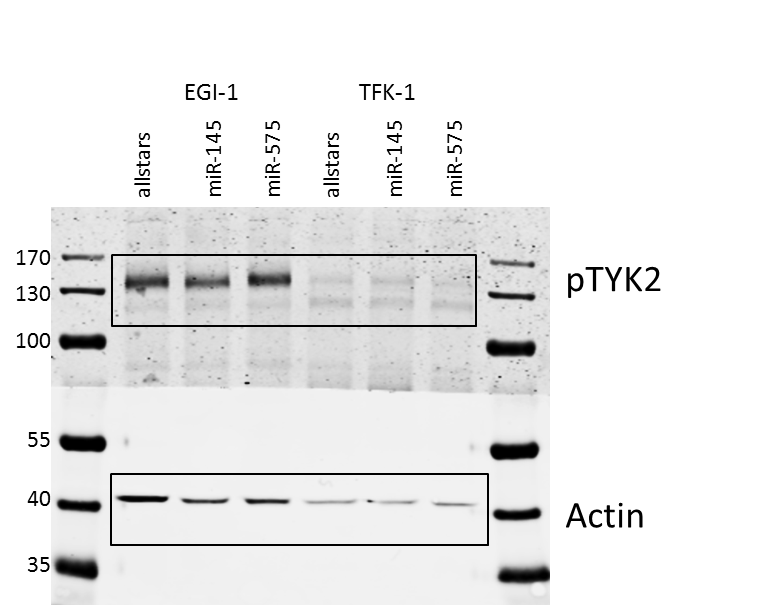
**

**Supplemental Figure S7C**

**
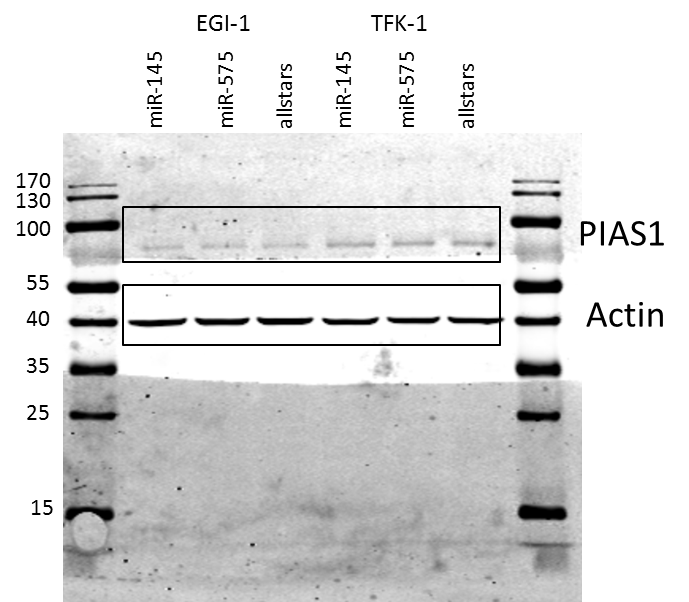
**

**
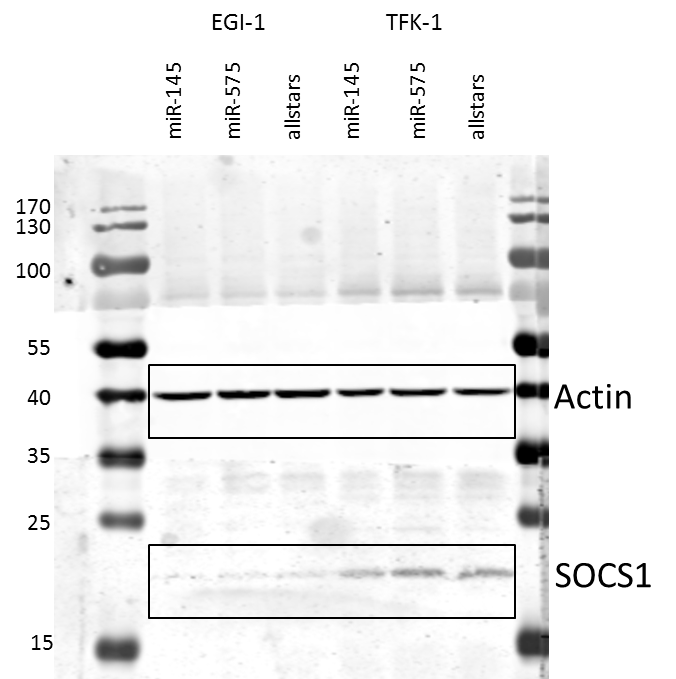
**

**Supplemental Figure S7D**

**
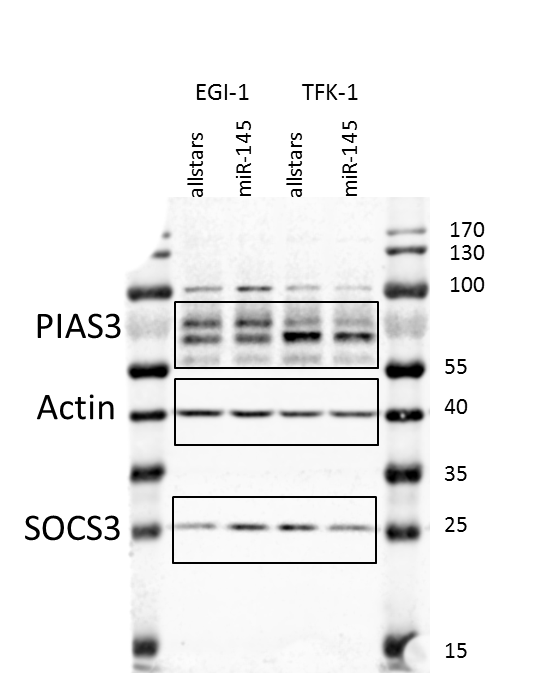
**

**
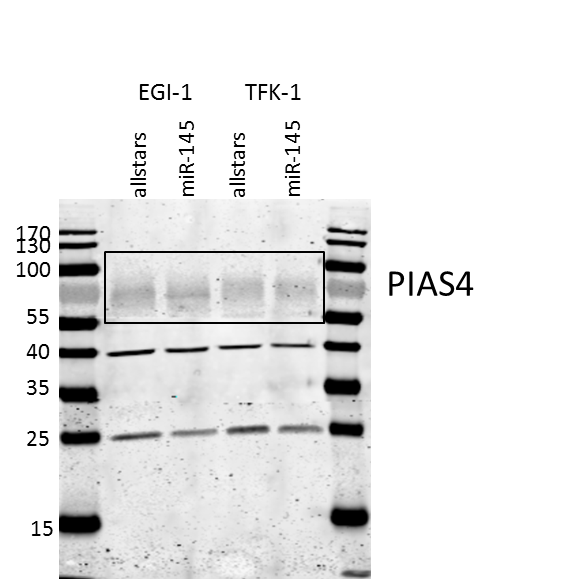
**

**Supplemental Tables**

**Table S1: Identification of survival-associated miRNAs in patients with short survival (Short; <18 months) and long survival (Long; >18 months) in comparison to non-neoplastic normal gallbladder tissue**

|  | **Mean expression^a^** | | | **Tumor vs Normal** | | | **Short vs Long** | |
| --- | --- | --- | --- | --- | --- | --- | --- | --- |
| **Probes** | **Normal** | **Long** | **Short** | **Difference** | **p-value^b^** | **adj. p** | **Difference** | **p-value** |
| **hsa-miR-338-3p** | 8.523 | 6.882 | 5.751 | -2.207 | 1.2E-04 | 4.3E-04 | -1.131 | 0.007 |
| **hsa-miR-145-5p** | 14.527 | 10.496 | 9.581 | -4.489 | 6.6E-11 | 1.2E-09 | -0.914 | 0.035 |
| **hsa-miR-29c-5p** | 8.209 | 6.198 | 5.794 | -2.213 | 9.3E-13 | 2.5E-11 | -0.405 | 0.029 |
| **hsa-miR-502-3p** | 6.472 | 5.627 | 5.371 | -0.973 | 1.5E-07 | 1.0E-06 | -0.256 | 0.046 |
| **hsa-miR-500a-3p** | 6.420 | 5.782 | 5.558 | -0.750 | 7.9E-07 | 4.6E-06 | -0.224 | 0.036 |
| **hsa-miR-103a-2-5p** | 4.954 | 4.825 | 4.734 | -0.174 | 2.8E-04 | 9.3E-04 | -0.090 | 0.011 |
| **hsa-miR-26a-1-3p** | 5.186 | 4.899 | 4.818 | -0.328 | 3.2E-08 | 2.7E-07 | -0.082 | 0.043 |
| **hsa-miR-188-3p** | 4.729 | 4.630 | 4.561 | -0.133 | 1.4E-04 | 5.0E-04 | -0.068 | 0.008 |
| **hsa-miR-4282** | 4.993 | 5.272 | 5.373 | 0.329 | 3.7E-07 | 2.3E-06 | 0.101 | 0.024 |
| **hsa-miR-3689f** | 5.205 | 5.828 | 5.932 | 0.675 | 8.7E-14 | 3.3E-12 | 0.104 | 0.049 |
| **hsa-miR-4300** | 4.892 | 5.068 | 5.207 | 0.245 | 2.8E-04 | 9.4E-04 | 0.139 | 0.005 |
| **hsa-miR-5587-5p** | 5.265 | 5.964 | 6.103 | 0.768 | 9.4E-12 | 2.0E-10 | 0.139 | 0.046 |
| **hsa-miR-5187-5p** | 5.137 | 5.427 | 5.571 | 0.362 | 1.4E-04 | 4.9E-04 | 0.144 | 0.042 |
| **hsa-miR-3689a-5p** | 4.957 | 5.447 | 5.608 | 0.571 | 3.3E-10 | 4.9E-09 | 0.161 | 0.005 |
| **hsa-miR-299-3p** | 6.206 | 5.664 | 5.863 | -0.442 | 5.4E-05 | 2.1E-04 | 0.199 | 0.013 |
| **hsa-miR-4502** | 5.508 | 6.181 | 6.383 | 0.774 | 3.8E-10 | 5.5E-09 | 0.202 | 0.011 |
| **hsa-miR-3156-5p** | 8.264 | 8.833 | 9.048 | 0.676 | 6.5E-06 | 3.1E-05 | 0.215 | 0.047 |
| **hsa-miR-4470** | 6.659 | 7.378 | 7.597 | 0.828 | 1.2E-08 | 1.1E-07 | 0.218 | 0.025 |
| **hsa-miR-4462** | 7.650 | 9.534 | 9.829 | 2.031 | 1.9E-17 | 2.5E-15 | 0.295 | 0.018 |
| **hsa-miR-4430** | 9.089 | 11.878 | 12.271 | 2.986 | 5.0E-18 | 1.1E-15 | 0.393 | 0.026 |
| **hsa-miR-6129** | 6.423 | 7.373 | 7.775 | 1.151 | 2.0E-07 | 1.3E-06 | 0.403 | 0.008 |
| **hsa-miR-125a-3p** | 8.854 | 10.176 | 10.607 | 1.537 | 7.8E-08 | 5.7E-07 | 0.430 | 0.028 |
| **hsa-miR-370** | 7.156 | 8.278 | 8.766 | 1.366 | 2.5E-05 | 1.0E-04 | 0.488 | 0.039 |
| **hsa-miR-575** | 10.129 | 10.970 | 11.544 | 1.128 | 2.4E-05 | 1.0E-04 | 0.573 | 0.003 |

^a^ log2 expression values; ^b^ Mann-Whitney U test

**Table S2: Fifty-eight most upregulated genes in TFK-1 cells transfected by miR-145-5p (adjusted p<0.05)**

| **Gene Name** | **Cytogenetic band** | **Control^a^** | **miR-145^a^** | **fold difference** | **adj. p-value^b^** | **STAT1 target^c^** |
| --- | --- | --- | --- | --- | --- | --- |
| IFI44L | 1p31.1 | 5.494 | 8.339 | 7.185 | 0.0002 |  |
| OAS2 | 12q24.2 | 5.210 | 7.813 | 6.078 | 0.0207 |  |
| IFITM1 | 11p15.5 | 7.665 | 10.195 | 5.776 | 0.0059 |  |
| MX1 | 21q22.3 | 6.730 | 8.855 | 4.363 | 0.0037 | Yes |
| MT2A | 16q13 | 8.250 | 10.055 | 3.495 | 0.0136 |  |
| OAS3 | 12q24.2 | 6.505 | 8.174 | 3.181 | 0.0058 |  |
| MT1JP | 16q13 | 6.926 | 8.531 | 3.041 | 0.0271 |  |
| EIF2AK2 | 2p22-p21 | 8.441 | 9.952 | 2.851 | 0.0184 |  |
| IFI6 | 1p35 | 12.035 | 13.515 | 2.789 | 0.0024 |  |
| ISG15 | 1p36.33 | 6.192 | 7.590 | 2.637 | 0.0248 |  |
| STAT1 | 2q32.2 | 7.827 | 9.184 | 2.561 | 0.0071 | Yes |
| IFITM2 | 11p15.5 | 8.052 | 9.373 | 2.498 | 0.0049 |  |
| OAS1 | 12q24.2 | 6.160 | 7.473 | 2.486 | 0.0039 |  |
| DDX60 | 4q32.3 | 5.311 | 6.603 | 2.447 | 0.0011 |  |
| PARP14 | 3q21.1 | 6.991 | 8.271 | 2.429 | 0.0061 |  |
| IFITM4P | 6p22.1 | 5.961 | 7.201 | 2.362 | 0.0024 |  |
| IFI27 | 14q32 | 10.828 | 12.067 | 2.361 | 0.0342 | Yes |
| IFI44 | 1p31.1 | 5.231 | 6.470 | 2.360 | 0.0012 |  |
| IFI35 | 17q21 | 6.441 | 7.668 | 2.341 | 0.0188 | Yes |
| PNPT1 | 2p15 | 5.927 | 7.097 | 2.250 | 0.0028 |  |
| LY6E | 8q24.3 | 10.787 | 11.954 | 2.245 | 0.0246 |  |
| DTX3L | 3q21.1 | 7.619 | 8.708 | 2.128 | 0.0116 | Yes |
| UBE2L6 | 11q12 | 6.984 | 7.975 | 1.987 | 0.0312 | Yes |
| PLSCR1 | 3q23 | 5.902 | 6.882 | 1.973 | 0.0101 | Yes |
| LOC100419583 | NA | 6.703 | 7.645 | 1.922 | 0.0065 |  |
| OASL | 12q24.2 | 5.491 | 6.407 | 1.886 | 0.0065 |  |
| HLA-B | 6p21.3 | 8.817 | 9.725 | 1.877 | 0.0101 |  |
| SAMHD1 | 20pter-q12 | 5.302 | 6.174 | 1.829 | 0.0162 |  |
| PARP9 | 3q21 | 5.654 | 6.488 | 1.782 | 0.0018 | Yes |
| USP18 | 22q11.21 | 5.930 | 6.704 | 1.710 | 0.0230 |  |
| LAP3 | 4p15.32 | 5.921 | 6.690 | 1.703 | 0.0032 | Yes |
| HLA-C | 6p21.3 | 9.132 | 9.874 | 1.673 | 0.0042 |  |
| IFIT1 | 10q23.31 | 5.056 | 5.795 | 1.669 | 0.0026 |  |
| TAP1 | 6p21.3 | 6.010 | 6.722 | 1.638 | 0.0012 | Yes |
| PSMB9 | 6p21.3 | 7.606 | 8.315 | 1.635 | 0.0136 | Yes |
| ADAR | 1q21.3 | 8.446 | 9.142 | 1.620 | 0.0271 |  |
| HIST3H2BB | 1q42.13 | 6.393 | 7.065 | 1.593 | 0.0188 |  |
| B2M | 15q21.1 | 11.948 | 12.611 | 1.583 | 0.0003 |  |
| MYEOV | 11q13 | 7.895 | 8.555 | 1.580 | 0.0141 |  |
| IFI16 | 1q22 | 5.254 | 5.884 | 1.547 | 0.0023 | Yes |
| SPATS2L | 2q33.1 | 7.445 | 8.068 | 1.540 | 0.0162 |  |
| IRF9 | 14q11.2 | 7.655 | 8.277 | 1.539 | 0.0192 | Yes |
| HLA-E | 6p21.3 | 6.372 | 6.973 | 1.517 | 0.0382 | Yes |
| AREG | 4q13.3 | 7.409 | 8.003 | 1.510 | 0.0486 |  |
| HLA-A | 6p21.3 | 9.218 | 9.802 | 1.499 | 0.0483 |  |
| LAMP3 | 3q26.3-q27 | 5.290 | 5.870 | 1.495 | 0.0018 |  |
| BST2 | 19p13.1 | 5.346 | 5.924 | 1.492 | 0.0014 | Yes |
| DDX60L | 4q32.3 | 5.444 | 6.020 | 1.491 | 0.0189 |  |
| HES1 | 3q28-q29 | 7.396 | 7.967 | 1.486 | 0.0463 |  |
| HCP5 | 6p21.3 | 5.962 | 6.522 | 1.474 | 0.0188 |  |
| HLA-H | 6p21.3 | 6.231 | 6.778 | 1.461 | 0.0463 |  |
| IFIT5 | 10q23.31 | 5.358 | 5.902 | 1.458 | 0.0008 |  |
| TRIM14 | 9q22.33 | 7.297 | 7.841 | 1.458 | 0.0026 |  |
| PSMB8 | 6p21.3 | 6.709 | 7.238 | 1.443 | 0.0033 | Yes |
| MX2 | 21q22.3 | 5.284 | 5.810 | 1.440 | 0.0044 |  |
| DDX58 | 9p12 | 5.282 | 5.788 | 1.421 | 0.0101 |  |
| PARP12 | 7q34 | 5.584 | 6.090 | 1.420 | 0.0158 |  |
| XAF1 | 17p13.1 | 5.222 | 5.725 | 1.417 | 0.0024 |  |

^a^ log2 normalized mean expression value; ^b^ T-test adjusted p-value; ^c^ reported as direct STAT1 target gene by Satoh, et al. Gene Regul Syst Bio, 2013; 7: 41–56

**Table S3: Thirty-one most down regulated genes in TFK-1 cells transfected by miR-145 (adjusted p<0.1)**

| **Gene Name** | **Cytogenetic band** | **Control^a^** | **miR-145^a^** | **fold difference** | **adj. p-value^b^** | **Predicted miR-145-5p target*** |
| --- | --- | --- | --- | --- | --- | --- |
| CRIP1 | 14q32.33 | 9.675 | 8.988 | 0.621 | 0.022 |  |
| DANCR | 4q12 | 6.488 | 5.948 | 0.688 | 0.026 |  |
| TSPAN1 | 1p34.1 | 6.867 | 6.350 | 0.699 | 0.069 |  |
| PTPRS | 19p13.3 | 7.777 | 7.279 | 0.708 | 0.034 |  |
| MUC5B | 11p15.5 | 9.456 | 8.961 | 0.710 | 0.083 |  |
| ALOX5 | 10q11.2 | 6.881 | 6.394 | 0.713 | 0.060 |  |
| PTPRF | 1p34 | 7.857 | 7.405 | 0.731 | 0.054 |  |
| ERBB3 | 12q13 | 7.645 | 7.204 | 0.737 | 0.061 |  |
| MVP | 16p11.2 | 7.014 | 6.594 | 0.747 | 0.087 |  |
| SULT2B1 | 19q13.3 | 8.249 | 7.849 | 0.758 | 0.021 |  |
| AHNAK2 | 14q32.33 | 6.795 | 6.396 | 0.758 | 0.019 |  |
| CMTM4 | 16q21-q22.1 | 7.274 | 6.883 | 0.763 | 0.002 | Yes |
| ANXA4 | 2p13 | 9.193 | 8.814 | 0.769 | 0.058 |  |
| AHNAK | 11q12.2 | 7.594 | 7.231 | 0.778 | 0.054 |  |
| SIAE | 11q24 | 5.963 | 5.601 | 0.778 | 0.026 |  |
| CACNG4 | 17q24 | 6.436 | 6.076 | 0.779 | 0.071 |  |
| QARS | 3p21.31 | 7.909 | 7.549 | 0.779 | 0.033 |  |
| RPS8 | 1p34.1-p32 | 11.228 | 10.876 | 0.784 | 0.053 |  |
| IMPDH2 | 3p21.2 | 7.876 | 7.531 | 0.788 | 0.049 |  |
| GNS | 12q14 | 6.405 | 6.062 | 0.788 | 0.063 | Yes |
| CHD3 | 17p13.1 | 6.796 | 6.456 | 0.790 | 0.006 |  |
| ACO1 | 9p21.1 | 5.777 | 5.442 | 0.793 | 0.035 |  |
| ITGB4 | 17q25 | 8.320 | 7.987 | 0.794 | 0.097 |  |
| IMPA2 | 18p11.2 | 8.053 | 7.729 | 0.799 | 0.033 |  |
| RPL12 | 9q34 | 10.039 | 9.715 | 0.799 | 0.021 |  |
| ACO2 | 22q13.2 | 9.634 | 9.311 | 0.800 | 0.050 |  |
| ARPC1B | 7q22.1 | 9.046 | 8.736 | 0.807 | 0.075 |  |
| ANXA9 | 1q21 | 10.462 | 10.153 | 0.807 | 0.002 |  |
| C12orf49 | 12q24.22 | 6.657 | 6.355 | 0.811 | 0.016 | Yes |
| PLEC | 8q24 | 6.801 | 6.499 | 0.811 | 0.019 |  |
| EEF2 | 19p13.3 | 11.321 | 11.020 | 0.811 | 0.023 |  |

^a^ log2 normalized expression value; ^b^ T-test adjusted p-value

* TargetScan ([www.targetscan.org/](http://www.targetscan.org/)) was used to search for predicted miR-145-5p target genes

**Table S4: KEGG pathway analysis of genes deregulated in miR145 expressing TFK-1 cells**

| **NAME** | **Sub Category** | **NES**^a^ | **p-value** | **q-value** | |
| --- | --- | --- | --- | --- | --- |
| Influenza A | 6.9 Infectious diseases: Viral | 2.382 | <0.001 | <0.001 |  |
| Measles | 6.9 Infectious diseases: Viral | 2.371 | <0.001 | <0.001 |  |
| Herpes simplex infection | 6.9 Infectious diseases: Viral | 2.343 | <0.001 | <0.001 |  |
| Autoimmune thyroid disease | 6.3 Immune diseases | 2.273 | <0.001 | <0.001 |  |
| Graft-versus-host disease | 6.3 Immune diseases | 2.223 | <0.001 | <0.001 |  |
| Antigen processing and presentation | 5.1 Immune system | 2.216 | <0.001 | <0.001 |  |
| Allograft rejection | 6.3 Immune diseases | 2.198 | <0.001 | <0.001 |  |
| Type I diabetes mellitus | 6.7 Endocrine and metabolic diseases | 2.176 | <0.001 | <0.001 |  |
| Hepatitis C | 6.9 Infectious diseases: Viral | 2.075 | <0.001 | 0.001 |  |
| Systemic lupus erythematosus | 6.3 Immune diseases | 1.981 | 0.002 | 0.003 |  |
| Viral myocarditis | 6.6 Cardiovascular diseases | 1.946 | <0.001 | 0.006 |  |
| Proteasome | 2.3 Folding, sorting and degradation | 1.850 | 0.004 | 0.020 |  |
| RIG-I-like receptor signaling pathway | 5.1 Immune system | 1.829 | 0.006 | 0.023 |  |
| NOD-like receptor signaling pathway | 5.1 Immune system | 1.801 | <0.001 | 0.029 |  |
| Viral carcinogenesis | 6.1 Cancers: Overview | 1.794 | <0.001 | 0.028 |  |
| Cytosolic DNA-sensing pathway | 5.1 Immune system | 1.784 | 0.004 | 0.029 |  |
| Steroid hormone biosynthesis | 1.3 Lipid metabolism | -1.622 | 0.006 | 0.047 |  |
| Insulin signaling pathway | 5.2 Endocrine system | -1.637 | <0.001 | 0.041 |  |
| Amino sugar and nucleotide sugar metabolism | 1.1 Carbohydrate metabolism | -1.656 | 0.002 | 0.034 |  |
| Drug metabolism - cytochrome P450 | 1.11 Xenobiotics biodegradation and metabolism | -1.665 | 0.004 | 0.034 |  |
| Retinol metabolism | 1.8 Metabolism of cofactors and vitamins | -1.675 | 0.002 | 0.031 |  |
| Metabolism of xenobiotics by cytochrome P450 | 1.11 Xenobiotics biodegradation and metabolism | -1.725 | <0.001 | 0.018 |  |
| Ascorbate and aldarate metabolism | 1.1 Carbohydrate metabolism | -1.731 | 0.002 | 0.018 |  |
| beta-Alanine metabolism | 1.6 Metabolism of other amino acids | -1.736 | <0.001 | 0.020 |  |
| Biosynthesis of amino acids | 1.0 Global and overview maps | -1.776 | <0.001 | 0.012 |  |
| Histidine metabolism | 1.5 Amino acid metabolism | -1.789 | 0.004 | 0.012 |  |
| Glyoxylate and dicarboxylate metabolism | 1.1 Carbohydrate metabolism | -1.861 | <0.001 | 0.004 |  |
| Starch and sucrose metabolism | 1.1 Carbohydrate metabolism | -1.915 | <0.001 | 0.002 |  |
| Peroxisome | 4.1 Transport and catabolism | -1.988 | <0.001 | 0.001 |  |
| Ribosome | 2.2 Translation | -2.012 | <0.001 | 0.001 |  |

^a^ NES: normalized enrichment score

**Table S5: Primer sequences and primer assays used in this study.**

| **Primer name** | **Sequence 5´- 3´** |
| --- | --- |
| STAT1 fw | ACCTAACGTGCTGTGCGTAG |
| STAT1 rev | GGTGAACCTGCTCCAGGAAT |
| MX1-fw | TGGCATAACCAGAGTGGCTG |
| MX1-rev | CCACATTACTGGGGACCACC |
| PARP9-fw | GGCCACATTGAATGGCAGAC |
| PARP9-rev | TACCAACTGGGACCGTTGAA |
| UBE2L6-fw | GGAACCTGTCCAGCGATGAT |
| UBE2L6-rev | TAGGGAGGTTGGTCGGGTAG |
| PTPRF-fw | CTGCGAACCTGTATGTGCGA |
| PTPRF-rev | CATCCACTTCACGTAGGGCA |
| ERBB3-fw | CATCGTGAGGGACCGAGATG |
| ERBB3-rev | GGAGCACAGATGGTCTTGGT |
| ERBB2-F1 | TGCTGGACATTGACGAGACAG |
| ERBB2-R1 | GTTTGGCCCCAAAAGTCATCA |
| Hs_miR-145_1 miScript Primer Assay |  |
| Hs_miR-575_1 miScript Primer Assay |  |
| Hs_miR-338_1 miScript Primer Assay |  |
| Hs_miR-370_1 miScript Primer Assay |  |
| Hs_SNORD48_11 miScript Primer Assay |  |
| Syn-hsa-mir-145-5p miScript miRNA Mimic | GUCCAGUUUUCCCAGGAAUCCCU |
| Syn-hsa-miR-575 miScript miRNA Mimic | GAGCCAGUUGGACAGGAGC |
| Syn-hsa-miR-338-3p miScript miRNA Mimic | UCCAGCAUCAGUGAUUUUGUUG |
| Syn-hsa-miR-370-3p miScript miRNA Mimic | GCCUGCUGGGGUGGAACCUGGU |
| AllStars Negative Control siRNA |  |

**Table S6: Antibodies used in this study.**

| Antigen | Species | Product/Company |
| --- | --- | --- |
| Actin | Mouse | 691001/MP Biomedicals |
| GAPDH | Chicken | AB2302/EMD Millipore |
| IRF7 | Rabbit | HPA052757/Atlas Antibodies |
| JAK1 | Rabbit | 3344/Cell Signaling |
| JAK2 | Rabbit | 3230/Cell Signaling |
| TYK | Rabbit | 9312/Cell Signaling |
| phospho-JAK1-Y1022 | Rabbit | 3331/Cell Signaling |
| phospho-JAK2-Y1007 | Rabbit | 3776/Cell Signaling |
| phospho-TYK-Y1054/1055 | Rabbit | 9321/Cell Signaling |
| phospho-STAT1-Ser727 (D3B7) | Rabbit | 8826/Cell Signaling |
| phospho-STAT1-Tyr701 (D4A7) | Rabbit | 7649/Cell Signaling |
| PIAS1 (D33A7) | Rabbit | 3550/Cell Signaling |
| PIAS3 (D5F9) | Rabbit | 9042/Cell Signaling |
| PIAS4 (D2F12) | Rabbit | 4392/Cell Signaling |
| PTPRF | Rabbit | LS-C408840/LifeSpan BioSciences |
| PTPRF/LAR | Rat | MAB3004/R&D Systems |
| SOCS1 (A156) | Rabbit | 3950/Cell Signaling |
| SOCS2 | Rabbit | 2779/Cell Signaling |
| SOCS3 (L210) | Rabbit | 2932/Cell Signaling |
| STAT1 (D1K9Y) | Rabbit | 14994/Cell Signaling |
| IRDye 680LT anti-mouse IgG | Donkey | 926-68022/LI-COR Biosciences |
| IRDye 800CW anti-mouse IgG | Donkey | 926-32212/LI-COR Biosciences |
| IRDye 800CW anti-rabbit IgG | Donkey | 926-32213/LI-COR Biosciences |
| IRDye 800CW anti-rat IgG | Goat | 926-32219/LI-COR Biosciences |
| IRDye 800CW anti-chicken IgG | Donkey | 926-32218/LI-COR Biosciences |
